# Chimeric antigen receptor T cells applied to solid tumors

**DOI:** 10.3389/fimmu.2022.984864

**Published:** 2022-10-31

**Authors:** Zhongguo Zhou, Can Tao, Jianting Li, Johnny Cheuk-on Tang, Albert Sun-chi Chan, Yuanyuan Zhou

**Affiliations:** ^1^ School of Chemistry and Molecular Biosciences, The University of Queensland, Brisbane, QLD, Australia; ^2^ School of Biomedical Engineering, Sun Yat-sen University, Guangzhou, Guangdong, China; ^3^ School of Clinical Medicine, Anhui Medical University, Hefei, Anhui, China; ^4^ Department of Applied Biology and Chemical Technology, The Hong Kong Polytechnic University, Hong Kong, Hong Kong SAR, China; ^5^ Kamford Genetics Company Limited, Hong Kong, Hong Kong SAR, China; ^6^ School of Pharmaceutical Sciences, Sun Yat-sen University, Guangzhou, Guangdong, China

**Keywords:** CAR T cell therapy, solid tumor, tumor microenvironment, antigenheterogeneity, crispr screens, neoantigen, progenitor-like T cell

## Abstract

Chimeric antigen receptor T cell (CAR-T) therapy is novel tumor immunotherapy that enables autologous T to express synthetic receptors to specifically recognize the surface tumor-associated antigens for exerting subsequent antitumor effects, and eliminating the resistance, metastases and recurrence of cancer. Although CAR T cells have exhibited success in eradicating hematologic malignancies, their applications to solid tumors has not yet been achieved due to obstacles such as the immune-suppressor tumor microenvironment and lack of tumor specific target antigens. In this review, we presented advancements in the development of CAR T cell therapy in solid tumors, and offered a brief summary of the challenges, as well as novel engineering and pharmaceutical interventions to overcome these barriers. Looking forward, we discussed the latest studies which are expected to reach the clinicals in the next few years, including CRISPR screens-based CAR modification and CAR T cells driven from progenitor-like T cells. Collectively, this review may inspire researchers and clinicians to develop clinical available strategies of CAR T cell therapies in solid tumor.

## Background

### Cancer immunotherapy

Cancer continues to be a major public health issue in the world. Researchers have estimated that there were around 19.3 million new cases of cancer and 10.0 million cancer deaths worldwide in 2020 (1). However, with the growing understanding of cancer cell characteristics and tumor subtypes classification, many kinds of cancer can be effectively treated by optimized chemo- or radiotherapy. Nevertheless, there is still room for improvement in cancer treatment due to the low survival rate caused by the high metastatic and relapse probability of some cancer subtypes and the excessive side effects of current therapies (2–4). There is ample evidence to support the critical role of the immune system in controlling and eradicating cancer, especially lymphocytes including natural killer (NK) cells that destroy compromised host cells in innate immunity, T cells involved in cell-mediated cytotoxic immunity, and B cells that participate in antibody-driven immunity (5, 6).

With the developments of biochemistry engineering and the deepening understanding of anti-tumor immunity mechanisms within the past few years, many advances have been made in the field of cancer immunotherapy. Cancer immunotherapy works by stimulating the immune system to improve pre-existing immunogenicity (e. g., cancer vaccines and adoptive cell therapy) or by neutralizing tumor-mediated immunosuppression (e. g., immune checkpoint blockade). One of the most successful immunotherapies so far is monoclonal antibodies (mAbs), which has been employed to enhance patients’ existing immune responses to cancer. There are over 100 mAbs approved by FDA for treating many diseases including several kinds of hematologic malignancies and solid tumors (7). These mAbs mainly induce tumor cell death through blocking the growth factor receptor signaling, and through engaging the antibody-dependent cellular phagocytosis (ADCP), complement-dependent cytotoxicity (CDC), or antibody-dependent cellular cytotoxicity (ADCC) (8).Among these mAbs, rituximab mainly acts through ADCC and CDC, was approved for treating Non-Hodgkin’s Lymphoma since 1997 (9). Recently, FDA approved rituximab in combination with chemotherapy to treat previously untreated pediatric patients (6 months to 18 years) with advanced stage of CD20-positive diffuse large B-cell lymphoma, Burkitt lymphoma, Burkitt-like lymphoma, or mature B-cell acute leukemia. At the interim analysis, the efficacy of rituximab was proved by the increased event-free survival events and decreased death events in the group receiving rituximab combinatory therapy (10). Inspired by the successful application of mAbs, other antibody formats such as bispecific antibodies and antibody derivatives have now been widely studied as alternative therapeutic agents for cancers (8).

Another example is immune checkpoint blockade (ICB), which has become the most popular strategy of cancer immunotherapy. Under normal conditions, the binding of inhibitory receptors to their ligands leads to the reduced T cell activation, giving rise to a counter-regulatory pathway during physiological T cell activation. Tumor cells utilize this counter-regulatory mechanism by activating inhibitory receptors such as CTLA-4 or PD-1 to deliver an inhibitory signal to T cells in order to escape immune elimination. However, treatment with ICB is able to downregulate this immune inhibitory function and shift the immune system balance toward T cell activation. Beginning with the approval of ipilimumab for treatment of melanoma in 2011, seven additional ICBs have been approved by the FDA for a broad range of different cancer indications (11). By combining anticancer drugs with PD-1 antibodies such as Nivolumab and Pembrolizumab, up to 41% of disease control rate was observed in metastatic triple-negative breast cancer (12). Therefore, the PD1/PDL1 pathway is now recognized as one of the most important checkpoints for immune responses, and PD1/PDL1-blckades alone or in combination with other therapies have become the first line treatment standard in many different cancers such as TNBC and metastatic melanoma (11).

### Chimeric antigen receptor T cells

**Figure 1:**
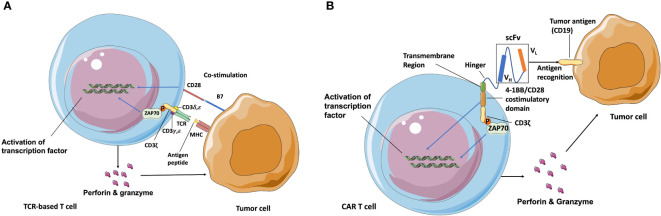
Schematic of TCR and CAR recognition and activation. **(A)** TCR is a heterodimer (one α and one β chain) connected by disulfide bonds that is expressed in complex with intracellular CD3 molecules (γ, δ, ζ, and ε). T cells are activated after TCR-recognizing peptides are presented by MHC molecules and co-stimulation of CD28 through B7 ligand. **(B)** CAR independently recognizes surface antigens with high affinity, unconstrained from MHC and co-stimulatory molecule expression on target cancer cells. The extracellular antigen-specific scFv is typically generated from connected V_H_ and V_L_ chains from mAbs against target antigens. Various hinges and transmembrane domains are employed to link scFv and intracellular signaling molecules, which are the co-stimulatory domain (4-1BB/CD28) and CD3ζ chain in second-generation CARs. CAR T cells are activated to release perforin, granzyme, and several kinds of cytokines after scFv recognition with surface antigens such as CD19 and from signaling through CD3ε-associated recruitment and the phosphorylation (P) of ZAP70 (13, 14). Blue arrow indicates the upregulation and activation of pathways. Black arrow indicates the molecules movement.

Adoptive T cell therapy (ATC) is another important type of cancer immunotherapy. Traditional ATCs rely on endogenous T cell repertoires which only recognize the major histocompatibility complex (MHC) molecules-presented antigens, and require co-stimulation of CD28 for activation (**Figure 1A**). While the genetically modified T cells can express CAR genes to recognize any surface antigen with high affinity independently of MHC molecules. During the manufacture of CAR T cells, leukocytes are generally harvested from patient’s blood *via* leukapheresis and enriched by counterflow centrifugal elutriation (15). The enriched T cells are then activated by stimulating antibodies in the presence of cytokines such as interleukin-2 (IL-2) IL-7, and IL-15 (16). What makes CAR T cells different from the traditional ATCs is the CAR construct, which is introduced into these enriched T cells after the activation step. A designed CAR construct encoding region is typically inserted into retroviral or lentiviral vectors and integrated into the T cell genome through transduction (17). After this, T cells are incubated in bioreactors for several days to express the CAR construct and then concentrated using a cell washer before infusion back into patients (18).

The first-generation of CARs were composed of the extracellular mAbs-derived variable domains and intracellular CD3ζ TCR-signaling chains, exhibiting the ability to recognize defined antigens and leading subsequent T cell activation, specific lysis, and cytokine release (19). However, due to the absence of co-stimulation domain, first-generation of CARs exhibited poor expansion and persistence, therefore had little efficacy in clinical trials (20). The second-generation of CAR T cells are regarded as the most mature generation in current clinical trials, and are composed of an extracellular single-chain variable fragment (scFv), a transmembrane spacer, and an intracellular co-stimulatory signaling domain followed by the intracellular region of CD3. The widely used co-stimulatory signaling domain such as CD3ζ plus 4-1BB or CD28 signaling domains could induce T cell expansion and longer persistence (21, 22). The extracellular scFv is typically derived from the variable light (V_L_) and variable heavy (V_H_) regions of a mouse-derived monoclonal antibody against target tumor antigens (23). The spacer/hinger domain links the scFv to the transmembrane region that across the cell membrane lipid bilayer (24) and steers the scFv domain to bind its cognate antigen through the flexibility of the hinger. Once the scFv portion recognizes and binds a tumor antigen, the intracellular 4-1BB or CD28 co-stimulatory domain and CD3ζ signal to the intracellular downstream pathway. The phosphorylated immunoreceptor tyrosine-based activation motif (ITAM) in CD3ζ domains then recruits the tyrosine kinase ζ-associated protein ZAP70, and leads to the downstream activation of NFAT, NF-κB, and AP-1 transcription factor families (25, 26) (**Figure 1B**). After that, activated T cells release perforin to punch holes at tumor cell membranes for the entry of cytotoxins and granzyme B to activate the apoptosis-related caspase cascades to initiate cell death. Currently, CAR structure has developed more and more complex forms. Various co-stimulatory domains beyond 4-1BB and CD28 (e. g., OX40, CD27, and ICOS) and alternative extracellular chain design (e.g., nanobodies derived from the variable domain of heavy chain-only antibodies can have comparable binding ability, specificity, and stability to scFv domain, and prevent undesired immune reactions against the linkers within scFv domain) have become the focus of clinical research (27). Moreover, the third-generation CAR T cells combines two co-stimulation domains in one CAR construct to enhance CAR T cells potency. The fourth-generation CAR T cells are additionally modified with an expression cassette containing a transgenic protein such as cytokine IL-12 to activate local immune reaction. They were generated to enhance the antitumor activity for the treatment of solid tumors and provide great potent in future CAR designs (28, 29).

After the first B cell leukemia patients received the second-generation of CD19-targeted CAR T cells and exhibited profound and lasting positive responses (20), several clinical studies also demonstrated the remarkable success of the second-generation CAR T cells in treating hematologic cancers. Among them, CAR T cells targeting the B lymphocyte antigen CD19 (Novartis’s tisagenlecleucel) showed exceptional success with a 83% overall remission rate in 63 patients with relapsed or refractory B cell precursor ALL (acute lymphoblastic leukemia) and received U.S. Food and Drug Administration (FDA) approval for the treatment of ALL, marking a historic first approval for CAR T cell therapy (30). Apart from four approved CD19-targeted CAR T cell therapies including lisocabtagene maraleucel (Breyanzi^®^), tisagenlecleucel (Kymriah^®^), axicabtagene ciloleucel (Yescarta^®^), and brexucabtagene autoleucel (Tecartus^®^), the first BCMA-directed CAR T cells idecabtagene vicleucel (Abecma^®^) was approved by the FDA for multiple myeloma on March 26, 2021 based on a 72% overall response rate and a 28% stringent complete response rate from a phase 2 KarMMa trial involving 127 patients with relapsed or refractory multiple myeloma (31). Recently, the FDA approved another BCMA-CAR T cells product ciltacabtagene autoleucel (CARVYKTI^®^) for the treatment of adult patients with relapsed or refractory multiple myeloma after four or more prior lines of therapy, with an overall response rate of 97.9% in efficacy evaluation (32, 33). Given such effectiveness showed in these cases, CAR T cells are expected to become the most popular method of treating B cell malignancies, and to have similar effects in treating other cancers such as solid tumors. Unfortunately, the current clinical results of CAR T cells in solid tumors are not as ideal as in hematologic cancers. In a study of 11 patients with EGFR-expressing advanced (>50% expression) relapsed/refractory nonsmall-cell lung cancer who received the EGFR-targeted CAR-T cells treatment, only 2 patients obtained partial responses (34). Nevertheless, this study is still one of the most positive trails for CAR T cell therapy in solid tumors. The reason for the different effectiveness of CAR T cell therapy in treating solid tumors and hematologic cancers is complex and has not been fully understood. In this review, we discussed the general factors affecting the efficacy and safety of CAR T cell therapy and some unique key barriers be confronted with in the treatment of solid tumors. Furthermore, we proposed possible solutions based on recent developments in CAR T cell therapies.

## Limitation and recent advances in CAR T cell therapies for solid tumors

The compelling success of CAR T cell therapy in treating hematologic malignancies has encouraged its development in solid tumors. Currently, there are more than 30 ongoing phase I or phase I/II clinical trials for CAR T cells treating several kinds of solid tumors (**Table 1**). For example, an anti-HER2 CAR T cells were validated to be effective at targeting HER2^+^ cancer in preclinical studies and are now being evaluated in a phase I/II clinical trial for treating HER2^+^ cancers including breast, colorectal, gastric, glioma, lung, ovarian, and pancreatic malignancies (NCT02713984) (42). However, many factors that are absent in hematologic malignancies, like trafficking obstacles and immunosuppressive environment, make CAR T cell therapy difficult to achieve sufficient treatment efficacy for solid tumors.

**Table 1:** Some current CAR T cell clinical trials for solid tumors (35–41).

Target antigen	Malignancies	Phase	NCT number
AXL/ROR2	Renal cell carcinoma	I/II	NCT03393936
CEA	Breast, colorectal, gastric, lung, and pancreatic	I	NCT02349724
Claudin18.2	Advanced gastric adenocarcinoma, pancreatic adenocarcinoma	I	NCT03159819
Claudin18.2	Solid tumors	I	NCT03874897
EGFR	EGFR positive malignancies (cholangiocarcinoma, colorectal, non–small cell lung cancer (NSCLC), ovarian, pancreatic, renal)	I/II	NCT01869166
EGFRvIII	Glioblastoma	I	NCT03726515
FAP	Mesothelioma	I	NCT01722149
Folate receptor-alpha	Ovarian, Fallopian tube cancer, primary peritoneal carcinoma	I	NCT03585764
GD2	Solid tumors	I/II	NCT02992210
GD2	Diffuse intrinsic pontine gliomas (DIPG), spinal diffuse midline glioma (DMG)	I	NCT04196413
GPC3	Lung squamous cell carcinoma	I	NCT02876978
HER2	Breast, colorectal, gastric, glioma, lung, ovarian, pancreatic	I/II	NCT02713984
HER2	HER-2 positive advanced solid tumors	I/II	NCT01935843
HER2	Metastatic malignant neoplasm in the brain or leptomeninges, HER2-positive breast cancer	I	NCT03696030
IL13Ra2	Recurrent or refractory malignant glioma	I	NCT02208362
Mesothelin	Breast, lung, malignant pleural disease, mesothelioma, metastases	I	NCT02414269
Mesothelin	Cervical, lung, mesothelioma, ovarian, pancreatic	I/II	NCT01583686
Mesothelin	Breast (triple negative), endometrial, mesothelioma, ovarian, pancreatic	I	NCT02580747
Mesothelin	Mesothelioma, metastatic pancreatic, ovarian	I	NCT02159716
Mesothelin	Pancreatic cancer	I	NCT03323944
Mesothelin	Metastatic HER2-negative breast cancer	I	NCT02792114
MUC1	Breast (triple negative), hepatocellular, NSCLC, pancreatic	I/II	NCT02587689
MUC1	Metastatic breast cancer	I	NCT04020575
MUC16	Advanced solid tumors	I	NCT02498912
PD1	Gastric, lung, liver	I/II	NCT02862028
PSCA	Castration-resistant prostate carcinoma, metastatic prostate carcinoma	I	NCT03873805
PSCA	Advanced solid tumors (pancreatic and prostate) expressing high levels of PSCA	I/II	NCT02744287
PSMA	Prostate Cancer	I	NCT03089203
ROR1	Breast (including triple negative), leukemias (ALL, CLL, mantle cell), NSCLC(estrogen, HER2/Neu, progesterone receptor negative)	I	NCT02706392
VEGFR2	Melanoma, metastatic cancer, renal	I/II	NCT01218867

In treating solid tumors, CAR T cell therapy has to face not only several similar challenges observed in hematologic malignancies, such as T cell exhaustion and CARs-related toxicity, but also many other barriers. For example, identifying tumor-specific antigens that are highly and uniformly expressed on solid tumors is considered to be the most challenging factor in CAR T cell design for treating solid tumors. Unlike the treatment of hematologic malignancies where the CD19-CAR T cells recognize the CD19^+^ tumor cells in the blood, CAR T cells must travel from the blood into solid tumor sites and infiltrate the stromal elements of the solid tumors. Even after successful infiltration, the tumor microenvironment (TME) of solid tumors often prevents the effective anti-tumor immune response from CAR T cells.

### CAR T cell expansion and exhaustion

Although the high degree of personalization makes it difficult to compare each approved CAR T cell product, clinical studies have shown that T cell expansion, persistence and memory phenotype are the key factors in the elimination of the cancer, whether in hematologic malignancies or solid tumors. Observed in first-generation CAR T cells, rapid CAR T cell exhaustion became the most significant and the first recognized factor that reduces CAR T cell efficacy (20). The enhanced expansion and persistence of second-generation CAR T cells provided evidence for the critical effect of co-stimulatory domains in CAR constructs (43). CAR T cells constructed with CD28 generally exhibit a more effector-like memory phenotype and undergo a more rapid expansion and subsequent decline, whereas 4-1BB CAR T cells have a more central memory phenotype and exhibit slower expansion but longer persistence (44) as 4-1BB co-stimulation signaling activates both glycolytic metabolism and fatty acid metabolism which enhance the cell cycle progression (45). Hence, 4-1BB-based CAR T cells are considered as a better option in CAR design to prevent CAR T cell exhaustion. However, when targeting tumor cells with low antigen density, 4-1BB-based CAR T cells sometimes fail to be activated, whereas this is not the case of CD28-based CAR T cells (46). Apart from 4-1BB and CD28 based CAR design, the combinatory design of co-stimulation domain in third-generation CAR T cells also works well to prevent T cell exhaustion. Zhou et al. showed superior efficiency of anti-CD19 CAR T cells that incorporate CD27-CD28 in a phase I trial treating B cell non-Hodgkin lymphomas. The overall response rate for the 21 patients was 67% with 43% of patients achieving a complete response (47). Additionally, Guedan et al. also explored ICOS in combination with 4-1BB co-stimulation, and found that this could effectively prevent the exhaustion of CAR T cells, and exhibit increased efficacy compared to second-generation CAR T cells in a mouse solid tumor model (48).

The expansion and exhaustion of CAR T cells also rely on the T cell subsets in the infused product, which determines the memory phenotype of the T cells. During current manufacture of CAR T cells, heterogeneous T cells consisting of both CD4^+^ and CD8^+^ T cells displaying an early memory phenotypes are used without subset separation. Researchers hypothesized that CAR T cells generated using certain subset of T cells such as central memory phenotype T cells (49), memory stem T cells (50), CD26^high^ T cells (51), and virus specific memory T cells (52) may exhibit greater replicative potential. Strong expansion potential and target-specific cytotoxicity have been documented in a T cell product composed of 50% central memory T cells and 46% stem cell-like memory T cells (50). Nevertheless, due to the low composition percentage of these subtypes in T cell populations, which makes them hard to separate from patients’ peripheral blood mononuclear cells (PBMC) and concentrate to desired levels, it is difficult to commercialize such CAR subtype T cells. Even though some methods to isolate defined T cell subsets under good manufacturing (GMP) conditions have been developed (53), the requirement for large-scale continuous and quantitative purification with GMP-quality reagents are still the great challenge to construct CAR T cells from these rarer subsets.

In addition, T cells can be modified or impacted by supplementary reagents during the ex vivo manufacture process to prevent their exhaustion after infusion. Shifrut et al. developed a CRISPR-based method coupled with single-cell RNA sequencing to identify key regulators of stimulation responses in T cells. The disruption of genes such as suppressors of cytokine signaling 1 (SOCS1), Tet methyl cytosine dioxygenase 2 (TET2), and transcription elongation factor B polypeptide 2 (TCEB2) have been shown to enhance T cell expansion and tumor-specific cytotoxicity (54). Modification of endogenous promoters and epigenetic can also be utilized to prevent T cell exhaustion (55). Wang et al. suggested that as a DNA methyltransferase inhibitor, decitabine, can be employed to reverse the DNA methylation that contributes to CAR T cell exhaustion (56). Moreover, the exogenous cytokines or pharmacological inhibitors used during the manufacturing process contribute to the exhaustion of CAR T cells. Zhou et al. compared the CAR T cells expanded from PBMCs using different cytokines and indicated that IL-2 expanded CAR T cells are more exhausted compared to IL-7/15-expanded ones (57). Watanabe et al. summarized that co-culturing of T cells with some pharmacological inhibitors (e.g., the IKT inhibitor ibrutinib, BET inhibitor JQ-1) can specifically suppress T cell exhaustion (58). All of these studies suggested that T cells modifications and cytokines/pharmacal supplementation may significantly improve the efficacy of the final infusion product.

### Cytokine release syndrome and neurotoxicity

**Figure 2:**
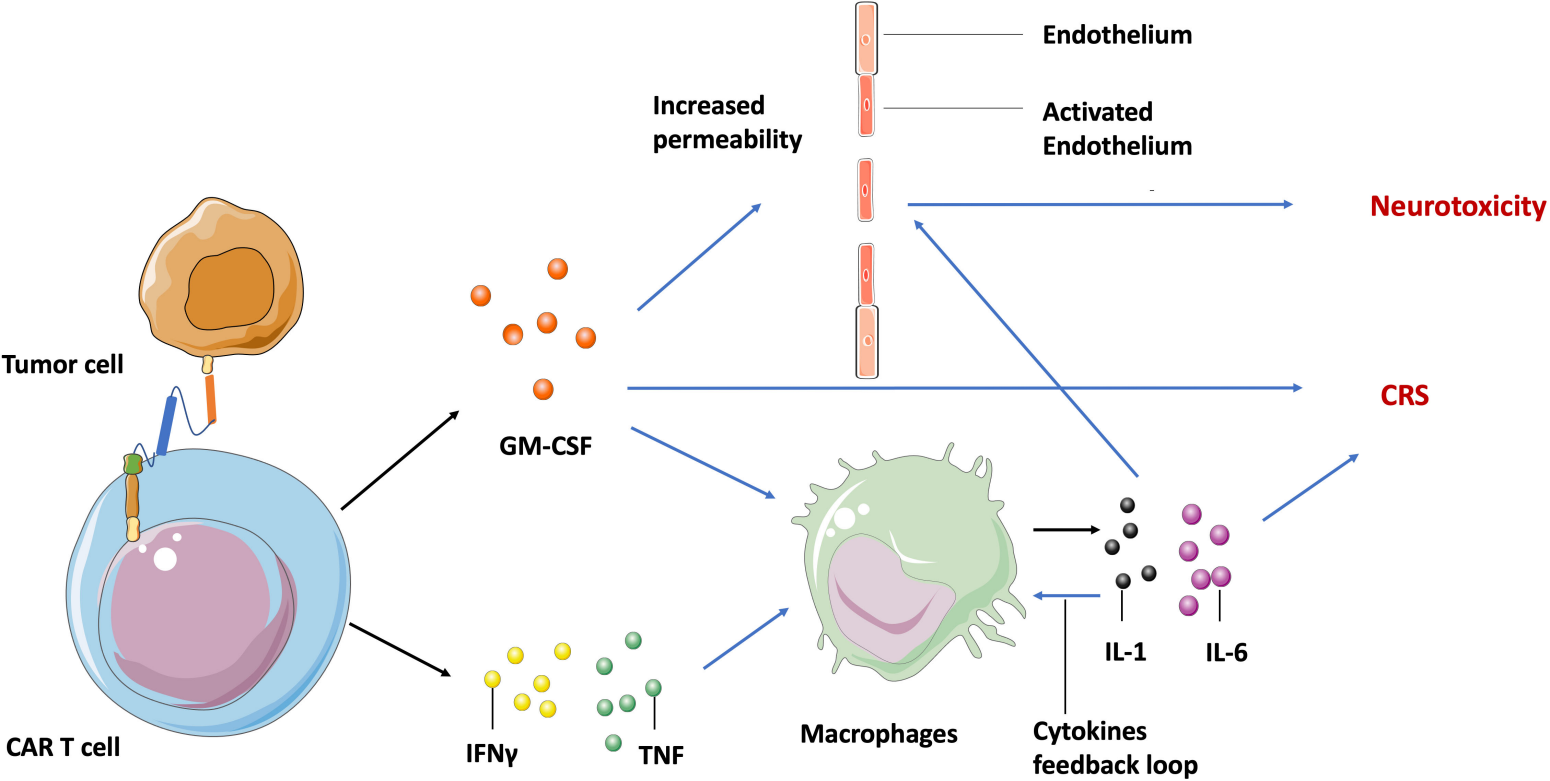
Molecular mechanism of CAR T cell-induced CRS and neurotoxicity. After recognizing tumor specific antigens, activated CAR T cells secret GM-CSF, TNF, and IFNγ, leading to the release of IL-1 and IL-6 by macrophages and other immune cells, which in turn activates macrophages to form a cytokine feedback loop. The GM-CSF and IL-6 may activate endothelium cells in the brain vasculature to increase its permeability. Blue arrow indicates the upregulation and activation of pathways. Black arrow indicates the molecules movement.

Despite the extreme potency of CAR T cell therapy, it has significant potential toxic side effects including cytokine release syndrome (CRS) and neurotoxicity in both hematologic malignancies and solid tumors. The manifestation spectrum of CRS ranges from fever, hypotension, to respiratory insufficiency (59), and neurotoxicity includes delirium, epileptic seizures, and even fatal cerebral edema (60). Both CRS and neurotoxicity appear frequently in clinical treatment and have been observed within CD19-specific, BCMA- specific T cells and EGFRvIII-CAR T cells during glioblastoma treatment (61). Additionally, CRS is associated with high circulating levels of several cytokines including granulocyte-macrophage colony-stimulating factor (GM-CSF), tumor necrosis factor (TNF), and interferon-γ (IFNγ) that are secreted by the activated CAR T cells. Theses cytokines then induce other immune cells, such as macrophages to release more cytokines (IL-1, IL-6), and create an inflammation loop called a cytokine storm (62) (**Figure 2**).

Although neurotoxicity is highly associated with systemic cytokine levels and CRS in clinical settings, the mechanisms involved are still not fully understood. Parker et al. found by single cell sequencing that some brain mural cells expressed CD19 on the cell surface. Hence, they hypothesized that neurotoxicity is probably caused by CD19-based CAR T cell activity against these cells (63). This may be an explanation for the off-target toxicity of CAR T cells, but can’t explain the neurotoxicity observed in patients treated with BCMA-specific T cells (64). Another generally accepted hypothesis is that inflammatory cytokines may activate endothelial cells in the brain vasculature, increasing vascular permeability to soluble mediators, CAR T cells, and other lymphocytes. The increased trafficking of cytokines and lymphocytes into central nervous system parenchyma then leads to focal edema and neurotoxicity (65) (**Figure 2**).

In most clinical cases, CRS and neurotoxicity are treatable and reversible. The most commonly used solution is to break the cytokine feedback loop by blocking the IL-6 receptor with mAb tocilizumab or inhibiting macrophage inflammatory activation to secrete IL-6 by using α-methyltyrosine (metyrosine). Other mAbs targeting GM-CSF (lenzilumab), IL-1 receptor (anakinra) and catecholamines (atrial natriuretic peptide), which induce CRS through a self-augmenting loop, are also employed to break the feedback loop in clinical settings (66, 67). Interventions to manage toxicity beyond the direct interruption of the cytokine loop are also under investigation, such as genetically altering CAR T cells to knock out the GM-CSF expressing gene (68), or introducing a suicide gene such as the inducible caspase-9 enzyme gene to get rid of CAR T cells within a short time after infusion (69).

### Antigen specificity

The primary reason for the success of CD19-based CAR design in eradicating hematologic malignancies is that CD19 is highly expressed on virtually all acute lymphoblastic leukemia cells, while other cells that express CD19 (B cells) are relatively discardable with the support of intravenous immunoglobulin (70). Despite the fact that BCMA- or CD22-targeted CAR T cells also exhibit tremendous anti-tumor activity in multiple myeloma and acute lymphoblastic leukemia (31, 71), all these target antigens are highly restricted to the B cell lineage. The ideal antigens in solid tumors should be selectively expressed at high level on all the tumor cells, but at a very low level or not at all on the surface of important normal tissues in order to improve safety. One study showed fatal toxicity shortly after the infusion of ERBB2/HER2- (a member of human epidermal growth factor receptor frequently overexpressed in many cancers) targeted CAR T cells, which was caused by the recognition and killing of ERBB2-positive cells expressed at low density on the lung endothelium and epithelium (72).

To prevent such on-target off-tumor toxicity, tumor-specific antigens that are only found in cancer cells are considered to be the ideal targets. Hence, CAR T cells targeting EGFR variant 3 (EGFRvIII), which is only expressed on malignant tumor cells (mostly glioblastomas), showed significant efficacy in treating mouse models of glioblastomas (73) and exhibited *in vivo* safety based on the current result of a phase I study at the University of Pennsylvania (NCT02209376) (61, 74). Aberrant Tn glycopeptides such as the Tn-glycosylated form of MUC1 and Tn-glycosylated podoplanin are another type of tumor-specific antigen that has attracted research interests recently. In particular, COSMC mutation observed in ovarian cancer, leukemia, breast cancer, sarcoma, and neuroblastoma leads to the Tn galactosylation of many surface peptides and therefore provides numerous potential targets for CAR T cell design (75). Posey et al. demonstrated the target-specific cytotoxicity of anti-Tn-MUC1 CAR T cells in xenograft models of leukemia and pancreatic cancer, and further proposed Tn glycosylated antigens as a novel class of targets (76).

Due to lack of more cancer-specific targets, most ongoing clinical trials of CAR T cell therapies for solid tumors continue to target tumor-associated antigens (TAAs) that are overexpressed in tumor cells but still expressed at low levels in normal tissues. One of these antigens is mesothelin (MSLN), which is a glycoprotein overexpressed in mesothelioma, ovarian, and pancreatic carcinomas but low expressed on the surface of peritoneal, pleural, and pericardial cells, has become an attractive target for CAR T cell therapy. One recent study has shown that MSLN-CAR T cells significantly decreased the MSLN^+^ tumor size in mouse models of colorectal and breast cancer (77). Initial results from a phase I clinical trial also suggest the safety and antitumor activity of MSLN-targeted CAR T cells (78). Despite the safety of this approach demonstrated in several clinical trials, the toxic potential of TAA-targeted CAR T cells should always be monitored carefully when changing any component of CAR T cells.

Richman et al. demonstrated lethal CNS toxicity induced by high-affinity GD2-targeted CAR T cells, which had not presented in previous GD2-targeted CAR T cells trials, and is possibly caused by excessive CAR T cells infiltration and proliferation within the brain (79). Consequently, it is critical to understand the expression levels of TAA in normal tissue in order to predict potential toxicity of TAA-targeted CAR T cells. To help with this, RNA-sequencing, microarrays, and immunohistochemistry are typically employed to detect certain antigens and analyze their expression levels. However, these technologies may underestimate the antigens expressed by very rare but critically important cells, or may fail to distinguish if expressed genes are derived from tested tissues or infiltrating cells (80). Single-cell RNA sequencing is a new technology that provides the expression profiles of individual cells and thus is able to distinguish target cells from infiltrating cells and other noncritical cells, enabling researchers to predict the toxic potential of novel TAA-targeted CAR T cells more effectively (81).

**Figure 3:**
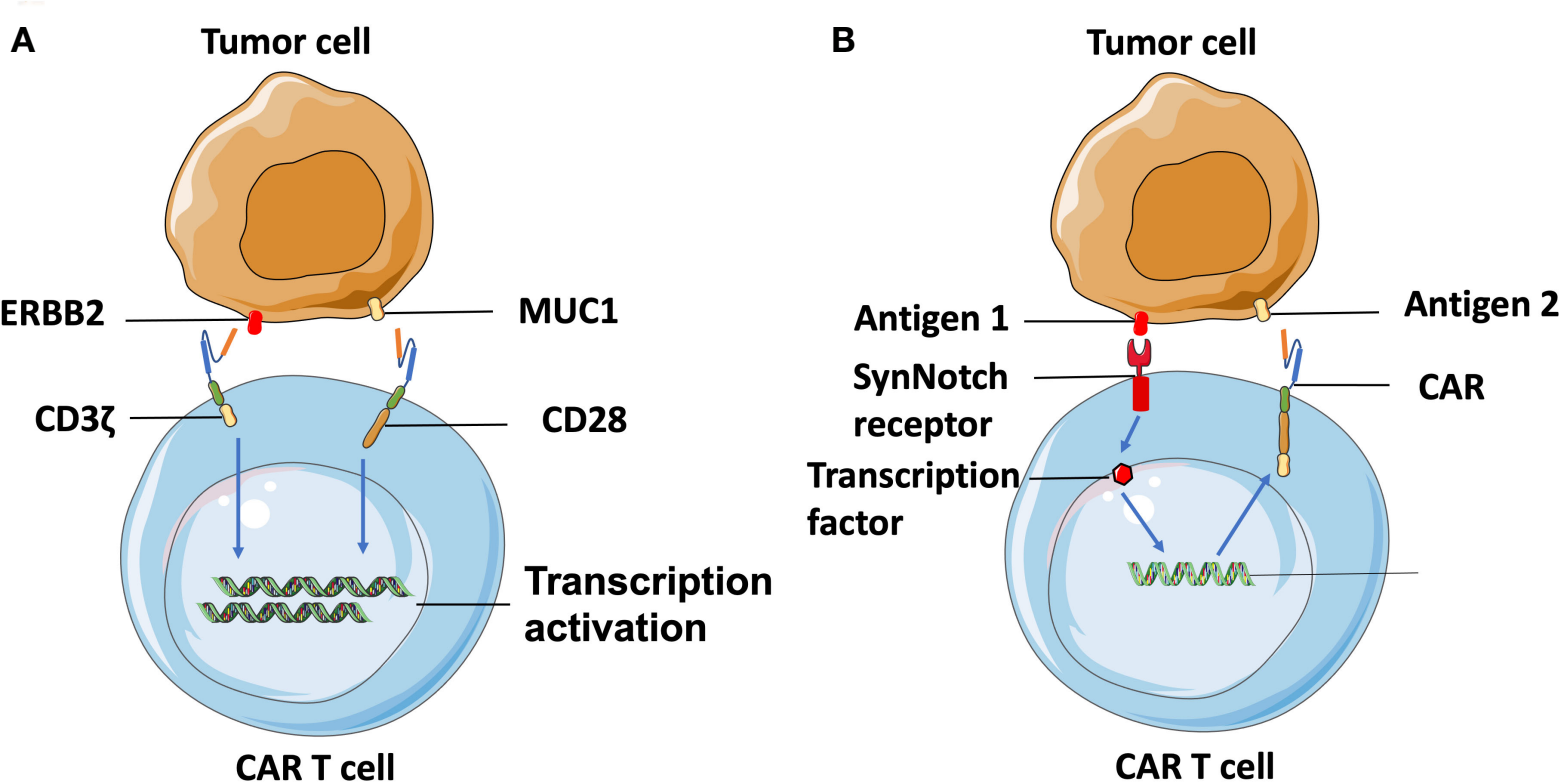
Combinatorial-antigen-targeted CAR. **(A)** ERBB2-targeted CAR signals the CD3ζ domain, and MUC1-targeted CAR signals the co-stimulatory CD28 domain. Hence, T cells can only be activated on the engagement of both HER2 and MUC1. **(B)** Binding of antigens with the extracellular domain of SynNotch receptors induces the proteolysis of the intracellular domain to produce a transcriptional regulator, which is subsequently translocated into the nucleus to regulate transcription of the gene encoding CAR. The CAR molecules are translated following recognition of the second antigen on the tumor cell, which can be the same cell expressing the first antigen or another, different type of cell.

Additionally, combinatorial antigen targeting is another novel approach developed for enhancing the specificity of TAA-targeted CAR T cells. Wikie et al. designed T cells co-expressing ERBB2- and MUC1-specific CAR that signals using CD3ζ and CD28 intracellular domains respectively and found that T cells targeting combinatorial antigens can only be fully activated in the presence of both antigens (82) (**Figure 3A**). A synthetic Notch receptor system has also been described in a combinatorically activated T cell. Recognized antigen firstly induces the transcription and expression of a CAR molecule for a second antigen, ensuring T cell activation only occurs in the presence of dual tumor antigens (83) (**Figure 3B**). Recently, combinatorial antigen targeting strategies were also considered in more advanced CAR designs, such as avidity-controlled CAR (84) and Adapter-CAR (85) to specifically eliminate tumors and protect essential tissues based on complex antigen expression profiles.

**Figure 4:**
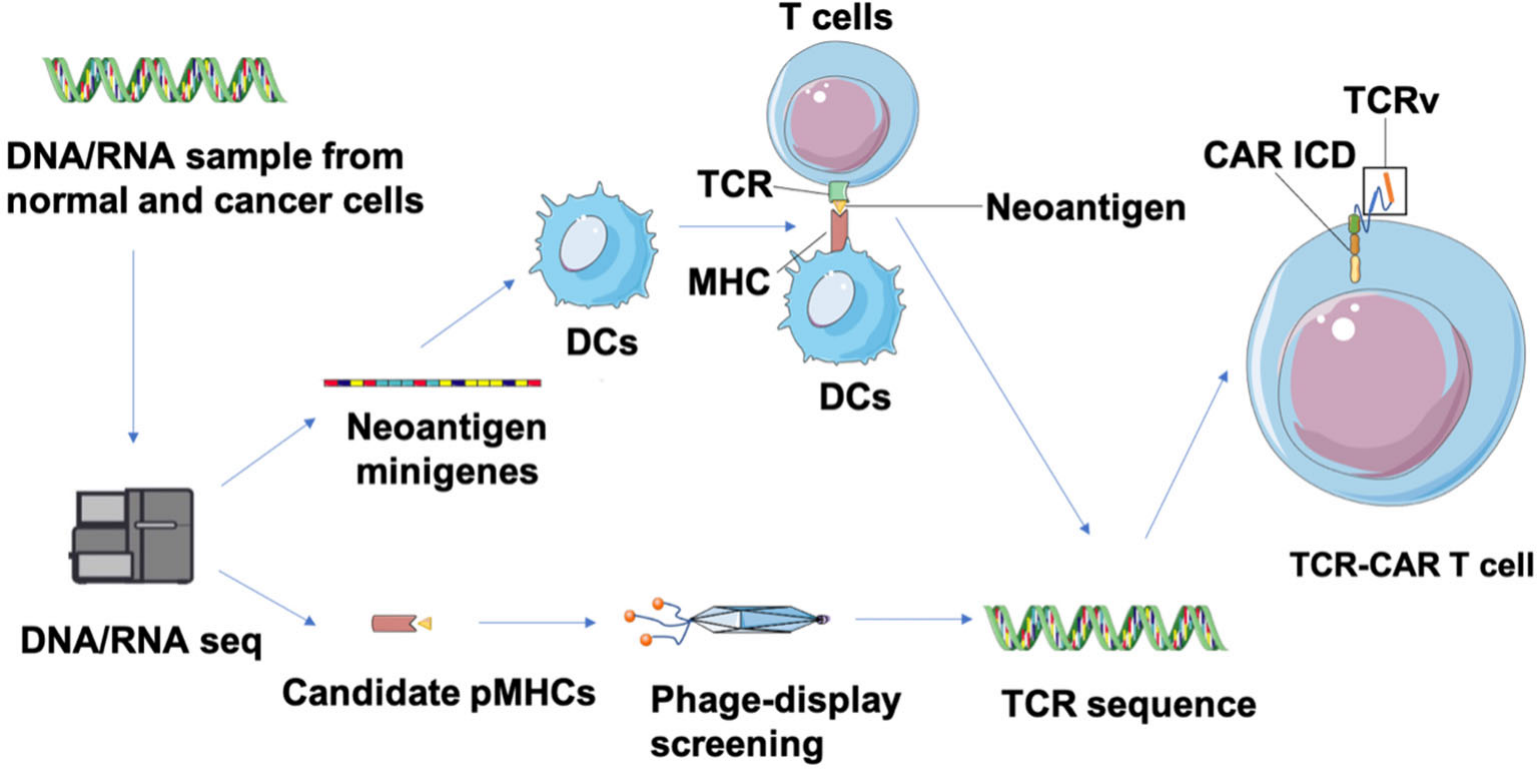
Identification of neoepitopes and development of neoantigen-specific TCR-CARs. Tumor and matched healthy tissue WES/RNA-seq is first performed to identity neoepitopes caused by NVM. Then, neoepitope pHMCs are employed for isolation of TCR-like antibodies through a phage-display screening. In parallel, neoantigen minigenes encoding potential neoepitopes can be designed and transfected into DCs that is co-cultured with T cells from healthy donors to obtain neoantigen-reactive T cells (89). Finally, a TCR sequence can be identified from isolated TCR-like antibodies and characterized neoantigen-reactive T cells. These TCRv genes can be used in a CAR structure to produce TCR-CAR T cells.

Neoantigens are caused by nonsynonymous mutations (NSM) in tumor of certain patients and are considered to be novel “tumor specific antigens” (86). Thus neoantigen-based immunotherapies have the potential to control tumors without on-target off-tumor toxicity in normal tissues. However, these neoantigens are located in the intracellular region and may only be displayed as antigenic peptides on cell surfaces through MHC class I or II molecules of cancer cells after intracellular degradation, which limits their target selection for CAR design. Fortunately, this limitation can be overcome by constructing TCR-CARs through engineering TCR-like antibody, which can recognize the peptides presented by MHC molecules (pMHCs) (87). First, tumor-specific NSM is generally characterized through whole-exome sequencing (WES) and RNA-seq (88) (**Figure 4**). Candidate neoepitopes are then selected based on strategies such as the pVACtools algorithm that predict their binding affinity to MHC molecules (88), and immunogenicity is evaluated through cytotoxicity assay to measure their actual potency toward neoepitope-reactive T cell response (89). Next, genes that encode the neoantigen-specific TCR β-chains or their variable regions are, either collected from TCR molecules generated by stimulated T cells co-cultured with neoantigens-expressing APC, or selected from the human scFV phage library screened against the neoepitopes (87). The genes encoding variable domains of TCR (TCRv) are then linked with the intracellular domain (ICD) of CARs to produce the TCR-CAR construct.

Contrary to conventional TCR T cells that may have low affinity to neoantigens and highly rely on the co-stimulation signal to be activated, TCR-CAR T cells enable independent, robust, and rapid activation (90). Recently, several neoantigen-specific TCR-CAR T cells have been constructed and validated for their anti-tumor efficacy. Walseng et al. designed two TCR-CAR-targeting neoantigens, MART-1 peptide26–35 (EAAGIGILTV) and TGFbR2 frameshift neoantigen peptide131–139 (RLSSCVPVA)30, and demonstrated their conserved specific binding ability to pMHCs (91). In another recent study, neoantigen NY-ESO-1-(SLLMWITQV) based TCR CAR T cells showed pMHC-specific induced activation and potent cytotoxicity to A375 cells loaded with NY-ESO-1 (92). Though neoantigen-based TCR-CARs can bypass the on-target toxicity occurring in TAA-based TCR-CARs, the risk for off-target toxicity of TCRs, especially TCRs with high affinity, remains a concern. Bijen et al. suggested a peptide library scan with a screening against more cell subsets to detect off-target toxicity of TCRs will benefit the preclinical testing (93). While most ongoing CAR T cell research is focused on TAA as the target, we suggest that TCR-CAR T cells that can recognize neoepitopes have remarkable potential for personalized cancer immunotherapy, especially for tumors with a high mutational burden such as melanoma.

### Antigen escape and heterogeneity

Antigen escape is a problem to long-term disease control with CAR-T cell therapy, and is considered to be the main mechanism associated with relapse in CAR T cell therapy, and has been widely observed in patients receiving CD19-CAR T cell treatment for hematological malignancies. Orlando et al. observed the loss of CD19 on the tumor cells surface through flow cytometry analysis of B-ALL cells from patients with relapsed B-ALL after CD19-targeted CAR T cell therapy, and identified the genetic mutations of the CD19 gene by sequencing of the genomes of these B-ALL cells (94). Selective pressure to mutate CD19 and outgrowth of pre-existing rare CD19-negative cells are considered as the major mechanisms of relapsed ALL cells escaping anti-CD19 CAR-T cells killing (95, 96). Furthermore, antigen escape has also been reported in solid tumor treatment and may be an even more serious challenge for solid tumors due their antigen heterogeneity.

**Figure 5:**
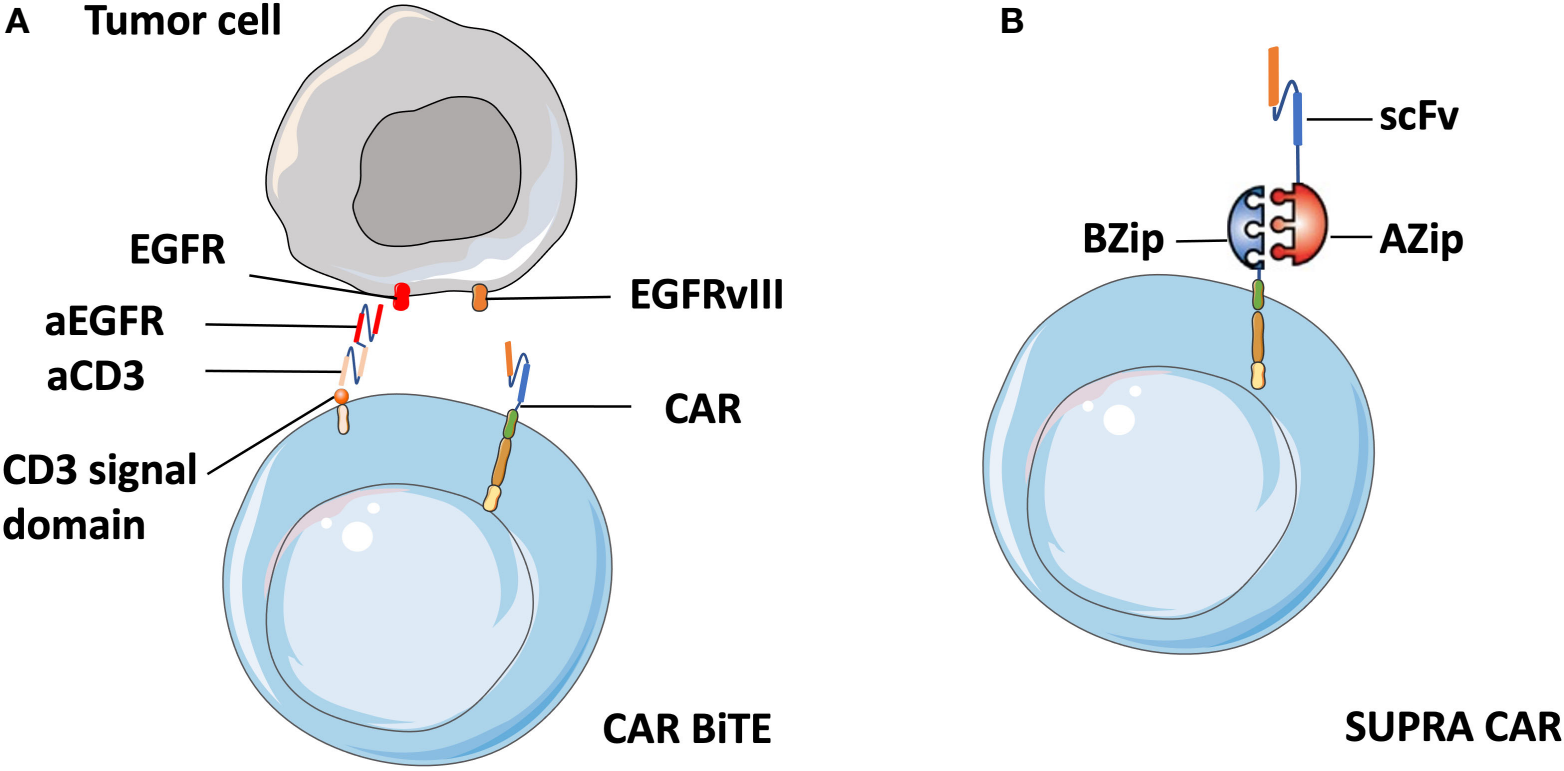
Schematic of CART.BiTE and SUPRA CAR. **(A)** Gene-modified EGFRvIII-CAR T cells deliver bispecific antibodies known as BiTEs6 that are combined with antibody against wild-type EGFR (aEGFR) and antibody against the CD3 signal domain (aCD3). Therefore, CART.BiTE cells obtain the capacity to target EGFRvIII-negative tumor cells. **(B)** A SUPRA CAR system is composed of a zipCAR and zipFv. A leucine zipper (BZip) serves as the extracellular portion of the CAR. A cognate leucine zipper that can bind to the BZip links with the scFv region.

Meenakshi et al. observed that exposing U373 cells (glioblastoma cell line) with HER2 expression to HER2-specific CAR T cells resulted in the emergence of HER2-negtive tumor cells (97). In a phase I clinical trial targeting glioblastoma patients, the infusion of EGFRvIII CAR-T cells led to the reduction of EGFRvIII expressing glioma cells. However, tumors that survived were still widespread and retained high levels of wild-type EGFR expression (61). To address antigenic heterogeneity and prevent antigen loss, the most straightforward approach is to develop CARs that target other antigens, either by modifying individual T cells with two distinct CAR molecules (98) or by constructing CAR molecules that contain two different binding domains in tandem (99). Different from the combinatorial antigen targeted CAR T cells that require the expression of two antigens together to activate T cells, either antigen input can trigger robust anti-tumor activity in dual-targeted CAR T cells. Fry et al. developed CD22–CD19-bispecific CAR T cells and demonstrated the killing effect of CD19^−^CD22^+^ and CD19^+^CD22^−^ B-ALL cell lines *in vitro*, as well as the significant B-ALL clearance in an NSG mouse model (99). Similarly, Choi et al. developed a bicistronic construct to drive expression of a EGFRvIII-specific CAR and a bispecific T cell engager (BiTE) against EGFR (61) based on their previous finding of viable tumors with wild-type EGFR expression, and showed their specific antitumor activity against heterogeneous solid tumors (100) (**Figure 5A**).

In addition, the quest for a split universal programmable (SUPRA) CAR system that can integrate signals from multiple antigens has recently become a flashpoint in CAR T cell therapy. A SUPRA CAR system is composed of a zipCAR that has a leucine zipper as the extracellular portion of the CAR and zipFv that has a scFv fused to a cognate leucine zipper that can bind to the leucine zipper on the zipCAR (101) (**Figure 5B**). Therefore, it can use different zipFvs to target multiple tumor antigens for the treatment of heterogeneous tumors and relapsed tumors with escaped antigens. Besides that, a SUPRA CAR system can finely tune T cell activation strength to mitigate over-activation by adjusting zipper affinity and zipFvs infusion levels. Thus we suppose that zipTCRv which targets numerous distinct neoantigens can be designed by combination of zipCAR construct design with neoantigen-specific TCR-CAR. Cytotoxic T cells with enhanced anti-tumor activity can then be redirected to tumor cells immediately after the infusion of zipTCRv, which may be useful in combatting relapse in solid tumors without re-engineering T cells.

### Trafficking obstacles

Unlike hematologic malignancies, CAR T cells must successfully target and infiltrate solid tumors, which depends on successful trafficking of the T cells from the blood into the solid tumor sites. Thus, local delivery of CAR T cells is now widely explored. Tchou et al. detected CAR mRNA in both peripheral blood and injected tumor tissues after intratumoral injection of mRNA c-Met-CAR T cells to c-Met-expressing breast cancer patients. They suggested that intratumoral injection were well tolerated and induced a detectable inflammatory response within tumors (102). Though intra-tumoral injection of CAR T cells can bypass trafficking obstacles and has been considered in several pre-clinical tests, it is unlikely to achieve systemic function under metastatic conditions (103, 104). The trafficking of immune cells generally depends on the matching between the chemokine receptors on the surface of the immune cells and the chemokines secreted by target cells, and adhesion receptors on both cells. Besides, CAR T cell trafficking is also impacted by the disorganized and uncoordinated expression of chemokines and their receptors within the TME.

CXCR3 is one kind of chemokine receptor, which is primarily expressed by T lymphocytes after T cell activation and is considered to be one of the most primal chemokine receptors on the surface of CAR T cells (105). Unfortunately, the mismatches of chemokines to their receptors, with the downregulated expression level of CXCR3 ligands in the TME in many cancers probably make CXCR3^high^ CAR T cells unable to effectively target tumor sites (106). To address this problem, CAR T cells that co-express better-matched chemokine receptors have recently been designed. Wang et al. constructed a mesothelin-specific CAR co-expressing cell chemokine receptors CCR2b and CCR4, and proved by trans-well assay that both CCR2b and CCR4 enhanced the migration of mesothelin-specific CAR T cells (107). Lin et al. also demonstrated greater anti-tumor activity of CCR2b-expressing T cells and increased infiltrated T cell numbers in a breast cancer model (108). The increased infiltration ability of chemokine-expressing T cells also addressed another significant physical obstacle during CAR- T cells trafficking, the blood-brain barrier (BBB). Recent research by Li et al. suggested that co-expression of CCR2b in B7-H3.CAR-T cells can improve migration toward CCL2 and the capability of passing BBB, providing enhanced antitumor activity against brain tumor lesions. They also proved the comparable antitumor activity of such CCR2b-B7-H3.CAR-T cells by co-culturing with NSCLC cell lines *in vitro*. Additionally, in A549 and H520 cancer brain co-xenograft tumor models, CCR2b co-expressing CAR-T cells showed superior antitumor activity through higher accumulation in the head region (109).

Moreover, as a kind of virus that specifically infects the tumor cells naturally, oncolytic viruses combined with chemotactic chemokines and cytokines both exhibited the potential to attract CAR T cells to tumor sites. In particular, IFN**γ**-mediated CXCL-9/10 secretion has been found to play a critical role in T cell trafficking within the TME (110). Based on the natural ability of oncolytic viruses to improve CXCL-9/10 expression levels locally within the TME, Fu et al. employed oncolytic viruses with adoptive T cells in pre-clinical studies and demonstrated that they facilitated T cell migration and persistence, leading to a significantly enhanced therapeutic effect (111). Arming oncolytic viruses with chemokine or cytokine ligands was considered to be an effective method to exploit their ability to reverse the inefficient trafficking of cytotoxic T cells.

Recently, Nishio et al. combined CAR T cells with an oncolytic virus armed with the chemokine RANTES and the cytokine IL15 in a neuroblastoma mouse model, and found that the intratumoral release of both RANTES and IL15 attracted CAR-T cells to improve CAR T cell influx (112). Moreover, the delivery of cytokines supporting APCs through oncolytic viruses may recruit DCs and modulate the cytokine milieu to facilitate an adaptive immune response (activated DCs can secrete CXCL-9/10), thus benefitting the CAR T cell trafficking, activation, and proliferation within the TME (113). Despite the fact that there are numerous potential strategies to combine oncolytic viruses and CAR T cell therapy, the complexity of oncolytic viruses and cellular interaction within the TME may lead to several unforeseen effects. For example, Ding et al. indicated that a certain isoform of chemokine receptor appears to drive tumor invasion in several types of cancers (114, 115), and Korniejewska et al. further suggested that the interaction of CXCR3-A (expressed on tumor cells) with CXCL-9 promotes tumor metastasis through activation of the phosphatidylinostol 3-kinase (PI3K) and mitogen-activated protein kinase (MAPK) signaling pathways. Due to the interaction of CXCR3-B, endothelial cell proliferation is downregulated, and tumor angiogenesis is depressed (116). Therefore, the complicated network between chemokines and their receptors in different immune cells puts forward further challenges for employing chemokine- armed oncolytic viruses with CAR T cells.

Apart from chemokine co-expression and oncolytic viruses, the rise of nanotechnology may also facilitate novel trafficking strategies. Nanomaterials have already been widely employed in many CAR T cell therapy pre-clinical trials, mainly for controlling the co-delivery of drugs and enable long-term retention with sustained exposure. Grosskopf et al. recently engineered a novel CAR T cell delivery platform based on Polymer-Nanoparticle hydrogels that induced a transient inflammatory niche for CAR-T cell expansion and activation in a mouse model, and provided a feasible treatment for metastases based on comparable efficacy during distal administration (117). Another character of nanoparticles that has not been fully explored when combining them with CAR T cell therapy is their modifiable surfaces. The modifiable surfaces and physicochemical features of nanomaterials also enable the specific trafficking of nanomaterial-based drugs, which has led to the recent production of several nanomaterial-based vaccines with target-specific trafficking abilities (118). The success of nanomaterial-based vaccine trafficking implies the potential for specifically trafficking of CAR T cells to the tumor sites of solid tumors in the future. However, there are still obstacles that need to be overcome in constructing nanomaterial-based T cell therapies, such as the toxicity caused by the oxidation and DNA destruction capacity of the nanomaterials (119). These obstacles, together with the concerns about the toxicity and applicability, make nanomaterial-based T cell therapies still far from being applied in the nowdays clinical trials.

### Immunosuppressive TME

The TME presents additional barriers to the successful application of CAR T cell therapy to solid tumors. The immunosuppressive TME contains multiple components, including physical, metabolic, and immunological barriers. Physical barriers are generated by stroma such as the carcinoma-associated fibroblasts, collagens, vascular beds, and dense extracellular matrix (ECM) with high tissue pressure to prevent extravasation (120). Genetically reprogrammed CAR T cells that secret enzymes, such as heparanase or hyaluronidase, that degrade ECM showed enhanced T cell penetration and better elimination of tumor cells (121). Another novel method of removing ECM involves the use of nanomaterials. Chen et al. encapsulated a photothermal agent, indocyanine green, into a PLGA nanomaterial and injected the encapsulated nanoparticle intratumorally at mice model. Subsequent near-infrared light irradiation allowed the photothermal agent to generate heat in the tumor site for the disruption of ECM before CAR T cell administration (122). Beside the enriched ECM, metabolic barriers such as hypoxia environment and lactate-induced acidosis, which are the consequence of excessive glycolysis of tumor cells, contribute to tumor genomic changes and metabolic dysfunction of immunogenic T cells (123). Interestingly, hypoxia environment can be employed by researchers as the selective sensor for CAR T cell activation. Kosti et al. proposed a rigorous hypoxia-sensing CAR T cell system that achieves selective expression of a pan-ErbB-targeted CAR molecules within the TME in solid tumor characterized by the hypoxia environment, achieving a considerable anti-tumor efficacy without off-tumor toxicity (124). To overcome such metabolic barriers, combination therapies such as applying hypoxia inducible factor antagonists with CAR T cells deserve consideration and further exploration.

Suppressive immune cells and cytokines also play the central role as the immunological barriers in mediating immune tolerance in tumor tissue. One promising strategy is to redirect or circumvent suppressive immune cells and cytokines. Transforming growth factor β (TGFβ) is one of the most important inhibitory tumor cytokines, which promotes epithelial-to-mesenchymal transition, could enhance ECM production, and directly suppress T cell effector functions (125). Several approaches have been developed to counteract the function of TGFβ in recent CAR T cell therapy studies. For example, the transduction of a dominant-negative TGFβ receptor (TGFβR) (126) or CRIPSR–Cas9-mediated knockout of the TGFβRII (127) has been found to induce increased efficacy and the central memory phenotype, as well as less exhaustion in xenograft solid tumor models.

Alternatively, it has been found that the secretion of pro-inflammatory soluble factors/cytokines through engineered CAR T cells or nanomaterials can increase the CAR T cells response and inhibit suppressor cells in the TME. Armored CAR constructs that overexpress cytokines such as IL-7, IL-12, and IL-18 have been shown to be able to activate more pro-inflammatory endogenous immune cells and exhibit enhanced anti-tumor response (128–130). Zhang et al. built lipid nanoparticles coated with the tumor-targeting peptide iRGD, and loaded with a combination of a PI3K inhibitor P-3065 and an α-GalCer agonist for therapeutic T cells, in order to transform the TME from suppressive to stimulatory state. In a mouse model of breast cancer, this lipid nanoparticle complex provided a two-week window for CAR T cells to home in on the lesion, undergo robust expansion, and trigger tumor regression (131).

Furthermore, combining CAR T cell therapy and ICB holds promise for counteracting the immunosuppressive TME and has been explored in several studies. This can be performed by cell-extrinsic strategies, in which ICB and CAR T cells are administered separately. Gargett et al. showed that the use of PD-1 blockade pembrolizumab together with GD2-CAR T cells enhanced T cell survival, and promoted killing activity in PD-L1^+^ tumor cell lines (132). The technique can also be performed using cell-intrinsic strategies, in which edited CAR T cells are employed alone without relying on repeated dosing of ICB. Cherkassky et al. constructed MSLN-specific third-generation CAR T cells with PD-1 dominant negative receptors that consisted of the extracellular domain of PD-1 without the intracellular signaling domain. It is proved that the co-transduction of such PD-1 receptors rescued CAR T cells from PD-1 ligand–mediated inhibition both *in vitro* and *in vivo* (133). More recently, Hu et al. designed an advanced delivery technology based on a “cell warehouse”, in which two different types of cells (human chondroitin sulfate proteoglycan 4 targeted CAR-T cells and platelets conjugated with PD-L1 ICB) and IL-15 encapsulated nanoparticles were loaded into an implantable hydrogel. The CAR-T cells could be released from the hydrogel slowly to avoid the TME- induced exhaustion of most CAR-T cells in a short time. In addition, modified platelets were activated and secreted anti-PD-L1 antibodies to block the immune checkpoint inhibition pathways and enhance the efficacy of the CAR-T cells (134).

## Future research

Despite CAR T cell therapy achieving remarkable outcomes and rapid development, many challenges discussed above have contributed to its limited success in solid tumors. These challenges must be overcome in order to get durable clinical benefits and maximize survival. Currently, driven by the interconnected feedback loops between clinical trials and lab-based research, CAR designs have shifted from simple permutations of co-stimulatory domains and scFv to more sophisticated strategies. For example, SUPRA CAR were developed to control CAR T cell activation remotely and CART.BiTE was designed to prevent off-tumor toxicity and antigen escape (135). Neoantigen-specific TCR-like CAR T cell therapy was proposed as a novel method to solve the biggest challenge in solid tumor T cell therapy, the on-target off-tumor toxicity from TAAs. Apart from neoantigen-CAR, further understanding about mechanisms in the TME and immune cells subpopulations, together with improved development of genetic approaches, are leading the way forward for increasing the efficacy of CAR T cell therapy in solid tumors.

### CRISPR screens and genetic modification

With improved understanding of key factors of intrinsic resistance mechanisms within TME, novel strategies targeting these factors using gene-editing and engineering approaches have been designed to enhance CAR T cell efficacy to clear out tumor cells and quash persistence within TME. The outsourced ex vivo designs and manufacture process of CAR T cells, different from other traditional drugs, also make it possible to modify the CAR T cells directly using genetic tools such as the CRISPR/Cas9 system. Besides the CRISPR/Cas9-mediated PD-1 knock out discussed above, Giuffrida et al. have indicated that targeting adenosine A_2A_ receptors with a clinically relevant CRISPR/Cas strategy can significantly increase the number of infiltrating CAR T cell and their ability to inhibit tumor growth. They hypothesized that this was driven by downregulated adenosine-mediated transcriptional pathways to enhance the production of IFNγ (136).

The indispensable function of IFNγ in CAR T cell therapy targeting solid tumors has also been demonstrated by Larson et al, who conducted a genome-wide CRISPR knockout screen in glioblastoma and found that the knout out of genes in the IFNγ receptor signaling pathway (e. g., IFNGR1, JAK1, and JAK2) made glioblastoma cells more resistant to CAR T cells both *in vitro* and *in vivo* (137). Such genome-wide systematic CRISPR screens may shift the CAR engineering from blindly focusing on individual features to combinations of multiple gene modifications with predicted benefits. Shifrut et al. and Legut et al. respectively performed a CRISPR/Cas9-based genome-scale screening, and identified negative regulators and synthetic drivers of T cell proliferation following stimulation by comparing the gene enrichment by barcode number after stimulation and incubation (54, 138). In addition, Ye et al. developed a dead-guide RNA-based genome scale gain-of-function CRISPR/Cas9 screen and identified PRODH2 as one of the key enzymes for reprogramming the proline metabolism in CAR-T cells, leading to enhanced antitumor efficacy (139). To conclude, CRISPR/Cas9 technology, especially genome-wide screens based on it, has accelerated the development of next-generation CAR-T cells and put forward exciting new directions for the design of therapeutic gene modification.

### Progenitor-like T cells

While current manufacturing efforts for adoptive cell therapy generally use CD4^+^/CD8^+^ T cell separation or just heterogeneous T cells obtained from certain patients, there is evidence to suggest that some subsets of T cells may be more efficacious as the source of CAR-T cells. However, it’s still unclear in which subpopulation of T cells the endowment of CAR will be most effective in treating certain cancers. Recently, several studies have identified that the less differentiated progenitor-like T cell subsets have a greater proliferative capacity (such as T memory stem cells (T_SCM_) and naïve T cells (T_N_)), which is a promising strategy to prolong the persistence of adoptive T cells and sustain remission (140). Based on a thorough paired analysis from high-dimensional flow cytometry, exome, single cell, and RNA sequencing, Ghorani et al. described the T cell differential landscape in TME and showed that gene feature redistribution from the progenitor-like state to differentiated dysfunctional states was highly associated with the unsatisfied prognosis of NSCLC (141).

Biasco et al. also demonstrated this finding in CAR T cell therapy clinically. They found that T_SCM_ instead of effector T cells became more prevalent over time with long-term maintenance of CAR T cell clones, suggesting the critical role of T_SCM_ cells, which account for only a small fraction in the infused product (142). Therefore, strategies to enhance the abundance or activity of the progenitor-like T cell pool in infused products may lead to therapeutic advantages. Nevertheless, the antitumor response potential of these T cell subpopulations after CAR engineering has not been fully explored, and require subset purification, CAR engineering, and validation. However, due to the limited sorting capacity and scalability of the current cell purification platforms, as well as the relative scarcity of circulating progenitor-T cells, the purification and enrichment of progenitor-T cells from PBMC samples has become the biggest manufacturing bottleneck impeding scale-up and scale-out efforts of this process (143).

An alternative method to produce progenitor-T cells that bypasses these limitations is to directly derive progenitor-T cells from hematopoietic stem and progenitor cells (HSPCs) or even from human pluripotent stem cells (hPSCs). Recently, Shukla et al. and Trotman et al. have designed the fully defined serum-free cell-free system, in which CD7+ progenitor-T cells can be differentiated from mouse or human HSPCs (144, 145). We proposed that a defined and scalable progenitor-T cell differentiation platform from hPSCs can be designed and further expanded by combining it with single cell sequencing to explore the evolutionary landscape of T cells and to identify the molecular switches for each T cell subpopulation.

## Conclusion

The tremendous success of CAR T cells in treating hematologic malignancies has led to increased commercial approvals in recent years. Now, the gradually-revealed toxicity and relapse potential of these treatments, together with their failure in solid tumors have sent researchers back to the proverbial drawing board. A better understanding of the mechanisms of efficacy, resistance, and solid tumor barriers has driven advances in both CAR engineering and clinical trial design. The novel delivery technologies such as cytokines encapsulated nanomaterials and cell warehouse have advanced the state of the art to overcome TME beyond the traditional methods. Innovations in CAR design like SUPRA CAR may very well lead to improved responses and may transform the treatment of solid tumors. Finally, we suggested that combining CAR T cell treatments with novel scRNA sequencing, CRISPR/Cas9-based screening and modification technology, neoantigen based TCR-CAR design, and CAR T cells driven from progenitor-like T cells may provide abundant potential treatment strategies for solid tumors and opportunities for additional improvements in the future.

## Author contributions

ZZ, YZ and AC was responsible for project design and writing of the manuscript, CT, JL and JT responsible for review, and editing. All authors contributed to the article and approved the submitted version.

## Conflict of interest

Author JT is employed by Kamford Genetics Company Limited.

The remaining authors declare that the research was conducted in the absence of any commercial or financial relationships that could be construed as a potential conflict of interest.

## Publisher's note

All claims expressed in this article are solely those of the authors and do not necessarily represent those of their affiliated organizations, or those of the publisher, the editors and the reviewers. Any product that may be evaluated in this article, or claim that may be made by its manufacturer, is not guaranteed or endorsed by the publisher.

## References

[1] Ferlay J Colombet M Soerjomataram I Parkin DM Piñeros M Znaor A. Cancer statistics for the year 2020: An overview. Int J Cancer (2021) 149:778–89. doi: 10.1002/ijc.33588 33818764

[2] Sailer V. Precision molecular pathology of prostate cancer. Cham: Springer International Publishing (2017) p. 279–95.

[3] Twardowski PW Figlin RA. Evidence-based urology. Chichester, UK: John Wiley & Sons, Ltd (2018) p. 399–405.

[4] Aydiner A. Breast disease. Cham: Springer International Publishing (2019) p. 463–94.

[5] Pandya PH Murray ME Pollok KE Renbarger JL. The immune system in cancer pathogenesis: Potential therapeutic approaches. J Immunol Res 2016 (2016) p:4273943. doi: 10.1155/2016/4273943 PMC5220497 28116316

[6] Huntington ND Cursons J Rautela J. The cancer–natural killer cell immunity cycle. Nat Rev Cancer (2020) 20(8):437–54. doi: 10.1038/s41568-020-0272-z 32581320

[7] Kaplon H Reichert JM. Antibodies to watch in 2021. MAbs (2021) 13(1):1860476. doi: 10.1080/19420862.2020.1860476 33459118 PMC7833761

[8] Jin S Sun Y Liang X Gu X Ning J Xu Y. Emerging new therapeutic antibody derivatives for cancer treatment. Signal Transduction Targeted Ther (2022) 7(1):39. doi: 10.1038/s41392-021-00868-x PMC8821599 35132063

[9] James JS Dubs G. FDA approves new kind of lymphoma treatment. food and drug administration. AIDS Treat News (1997) 1997(No 284):2–3. 11364912

[10] Minard-Colin V Aupérin A Pillon M Burke GAA Barkauskas DA Wheatley K. Rituximab for high-risk, mature b-cell non-hodgkin's lymphoma in children. N Engl J Med (2020) 382(23):2207–19. doi: 10.1056/NEJMoa1915315 PMC7720281 32492302

[11] Korman AJ Garrett-Thomson SC Lonberg N. The foundations of immune checkpoint blockade and the ipilimumab approval decennial. Nat Rev Drug Discovery (2022) 21(7):509–28. doi: 10.1038/s41573-021-00345-8 34937915

[12] Nanda R Chow LQM Dees EC Berger R Gupta S Geva R. Pembrolizumab in patients with advanced triple-negative breast cancer: Phase ib KEYNOTE-012 study. J Clin Oncol Off J Am Soc Clin Oncol (2016) 34(21):2460–7. doi: 10.1200/JCO.2015.64.8931 PMC6816000 27138582

[13] Almåsbak H Aarvak T Vemuri MC. CAR T cell therapy: A game changer in cancer treatment. J Immunol Res 2016 (2016) p:5474602–10. doi: 10.1155/2016/5474602 PMC4889848 27298832

[14] June CH O'Connor RS Kawalekar OU Ghassemi S Milone MC. CAR T cell immunotherapy for human cancer. Science (2018) 359(6382):1361–5. doi: 10.1126/science.aar6711 29567707

[15] Maus MV June CH Thomas AK Leonard DGB Schlienger K Allman D. Ex vivo expansion of polyclonal and antigen-specific cytotoxic T lymphocytes by artificial APCs expressing ligands for the T-cell receptor, CD28 and 4-1BB. Nat Biotechnol (2002) 20(2):143–8. doi: 10.1038/nbt0202-143 11821859

[16] Gong W Hoffmann J-M Stock S Wang L Liu Y Schubert M-L. Comparison of IL-2 vs IL-7/IL-15 for the generation of NY-ESO-1-specific T cells. Cancer Immunol Immunother (2019) 68(7):1195–209. doi: 10.1007/s00262-019-02354-4 PMC11028180 31177329

[17] Milone MC O'Doherty U. Clinical use of lentiviral vectors. Leukemia (2018) 32(7):1529–41. doi: 10.1038/s41375-018-0106-0 PMC6035154 29654266

[18] Levine BL. Performance-enhancing drugs: Design and production of redirected chimeric antigen receptor (CAR) T cells. Cancer Gene Ther (2015) 22(2):79–84. doi: 10.1038/cgt.2015.5 25675873

[19] Hwu P Yang JC Cowherd R Treisman J Shafer GE Eshhar Z. In vivo antitumor activity of T cells redirected with chimeric antibody/T-cell receptor genes. Cancer Res (1995) 55(15):3369–73. 7614473

[20] Michael HK Jennifer AW Linda LP Gang W Zelig E Sharon AM. A phase I study on adoptive immunotherapy using gene-modified T cells for ovarian cancer. Clin Cancer Res (2006) 12(20):6106–15. doi: 10.1158/1078-0432.CCR-06-1183 PMC2154351 17062687

[21] Kochenderfer JN Wilson WH Janik JE Dudley ME Stetler-Stevenson M Feldman SA. Eradication of b-lineage cells and regression of lymphoma in a patient treated with autologous T cells genetically engineered to recognize CD19. Blood (2010) 116(20):4099–102. doi: 10.1182/blood-2010-04-281931 PMC2993617 20668228

[22] Porter DL Levine BL Kalos M. Chimeric Antigen Receptor-Modified T Cells in Chronic Lymphoid Leukemia (vol 365, pg 725, 2011). New Engl J Med (2016) 374(10):998–8. doi: 10.1056/NEJMoa1103849 PMC3387277 21830940

[23] Wagner DL Fritsche E Pulsipher MA Ahmed N Hamieh M Hegde M. Immunogenicity of CAR T cells in cancer therapy. Nat Rev Clin Oncol (2021) 18:379–93. doi: 10.1038/s41571-021-00476-2 PMC8923136 33633361

[24] Brudno JN Lam N Vanasse D Shen Y-W Rose JJ Rossi J. Safety and feasibility of anti-CD19 CAR T cells with fully human binding domains in patients with b-cell lymphoma. Nat Med (2020) 26(2):270–80. doi: 10.1038/s41591-019-0737-3 PMC7781235 31959992

[25] Feucht J Sun J Eyquem J Ho Y-J Zhao Z Leibold J. Calibration of CAR activation potential directs alternative T cell fates and therapeutic potency. Nat Med (2019) 25(1):82–8. doi: 10.1038/s41591-018-0290-5 PMC6532069 30559421

[26] Gallagher MP Conley JM Vangala P Garber M Reboldi A Berg LJ. Hierarchy of signaling thresholds downstream of the T cell receptor and the tec kinase ITK. Proc Natl Acad Sci (2021) 118(35):e2025825118. doi: 10.1073/pnas.2025825118 34452995 PMC8536361

[27] Safarzadeh Kozani P Naseri A Mirarefin SMJ Salem F Nikbakht M Evazi Bakhshi S. Nanobody-based CAR-T cells for cancer immunotherapy. biomark Res (2022) 10(1):24. doi: 10.1186/s40364-022-00371-7 35468841 PMC9036779

[28] Chmielewski M Abken H. TRUCKs: the fourth generation of CARs. Expert Opin Biol Ther (2015) 15(8):1145–54. doi: 10.1517/14712598.2015.1046430 25985798

[29] Till BG Jensen MC Wang J Qian X Gopal AK Maloney DG. CD20-specific adoptive immunotherapy for lymphoma using a chimeric antigen receptor with both CD28 and 4-1BB domains: pilot clinical trial results. Blood (2012) 119(17):3940–50. doi: 10.1182/blood-2011-10-387969 PMC3350361 22308288

[30] Mullard A. FDA Approves first CAR T therapy. Nat Rev Drug Discovery (2017) 16(10):669–9. doi: 10.1038/nrd.2017.196 28959944

[31] Ned P. FDA Approves first CAR-T cell therapy for multiple myeloma. HR Dive (2021).

[32] Berdeja JG Madduri D Usmani SZ Jakubowiak A Agha M Cohen AD. Ciltacabtagene autoleucel, a b-cell maturation antigen-directed chimeric antigen receptor T-cell therapy in patients with relapsed or refractory multiple myeloma (CARTITUDE-1): a phase 1b/2 open-label study. Lancet (2021) 398(10297):314–24. doi: 10.1016/S0140-6736(21)00933-8 34175021

[33] Mullard A. FDA Approves second BCMA-targeted CAR-T cell therapy. Nat Rev Drug Discovery (2022) 21(4):249. doi: 10.1038/d41573-022-00048-8 35277677

[34] Feng K Guo Y Dai H Wang Y Li X Jia H. Chimeric antigen receptor-modified T cells for the immunotherapy of patients with EGFR-expressing advanced relapsed/refractory non-small cell lung cancer. Sci China. Life Sci (2016) 59(5):468–79. doi: 10.1007/s11427-016-5023-8 26968708

[35] Zeltsman MDO Dozier JMD McGee EBS Ngai DBS Adusumilli PSMDFF. CAR T-cell therapy for lung cancer and malignant pleural mesothelioma. Transl Res (2017) 187:1–10. doi: 10.1016/j.trsl.2017.04.004 28502785 PMC5581988

[36] Majzner RG Ramakrishna S Yeom KW Patel S Chinnasamy H Schultz LM. GD2-CAR T cell therapy for H3K27M-mutated diffuse midline gliomas. Nature (2022) 603(7903):934–41. doi: 10.1038/s41586-022-04489-4 PMC8967714 35130560

[37] Narayan V Barber-Rotenberg JS Jung I-Y Lacey SF Rech AJ Davis MM. PSMA-targeting TGFβ-insensitive armored CAR t cells in metastatic castration-resistant prostate cancer: a phase 1 trial. Nat Med (2022) 28(4):724–34. doi: 10.1038/s41591-022-01726-1 PMC10308799 35314843

[38] Zhan X Wang B Li Z Li J Wang H Chen L. Phase I trial of claudin 18.2-specific chimeric antigen receptor T cells for advanced gastric and pancreatic adenocarcinoma. J Clin Oncol (2019) 37(15_suppl):2509–9. doi: 10.1200/JCO.2019.37.15_suppl.2509

[39] Guo Y Feng K Liu Y Wu Z Dai H Yang Q. Phase I study of chimeric antigen receptor-modified T cells in patients with EGFR-positive advanced biliary tract cancers. Clin Cancer Res (2018) 24(6):1277–86. doi: 10.1158/1078-0432.CCR-17-0432 29138340

[40] Feng K Liu Y Guo Y Qiu J Wu Z Dai H. Phase I study of chimeric antigen receptor modified T cells in treating HER2-positive advanced biliary tract cancers and pancreatic cancers. Protein Cell (2018) 9(10):838–47. doi: 10.1007/s13238-017-0440-4 PMC6160389 28710747

[41] Patel U Abernathy J Savani BN Oluwole O Sengsayadeth S Dholaria B. CAR T cell therapy in solid tumors: A review of current clinical trials. eJHaem (2022) 3(S1):24–31. doi: 10.1002/jha2.356 35844304 PMC9175685

[42] Yang Z. A clinical research of CAR T cells targeting HER2 positive cancer. Bethesda, Maryland: ClinicalTrials.gov (2016).

[43] van der Stegen SJC Hamieh M Sadelain M. The pharmacology of second-generation chimeric antigen receptors. Nat Rev Drug Discovery (2015) 14(7):499–509. doi: 10.1038/nrd4597 26129802 PMC6410718

[44] Long AH Haso WM Shern JF Wanhainen KM Murgai M Ingaramo M. 4-1BB costimulation ameliorates T cell exhaustion induced by tonic signaling of chimeric antigen receptors. Nat Med (2015) 21(6):581–90. doi: 10.1038/nm.3838 PMC4458184 25939063

[45] Choi BK Lee DY Lee DG Kim YH Kim S-H Oh HS. 4-1BB signaling activates glucose and fatty acid metabolism to enhance CD8 + T cell proliferation. Cell Mol Immunol (2017) 14(9):748–57. doi: 10.1038/cmi.2016.02 PMC5596242 26972770

[46] Majzner RG Rietberg SP Sotillo E Dong R Vachharajani VT Labanieh L. Tuning the antigen density requirement for CAR T-cell activity. Cancer Discovery (2020) 10(5):702–23. doi: 10.1158/2159-8290.CD-19-0945 PMC7939454 32193224

[47] Zhou X Tu S Wang C Huang R Deng L Song C. Phase I trial of fourth-generation anti-CD19 chimeric antigen receptor T cells against relapsed or refractory b cell non-Hodgkin lymphomas. Front Immunol (2020) 11:564099–9. doi: 10.3389/fimmu.2020.564099 PMC7731732 33329526

[48] Guedan S Posey AD Shaw C Wing A Da T Patel PR. Enhancing CAR T cell persistence through ICOS and 4-1BB costimulation. JCI Insight (2018) 3(1). doi: 10.1172/jci.insight.96976 PMC5821198 29321369

[49] Wang X Popplewell LL Wagner JR Naranjo A Blanchard MS Mott MR. Phase 1 studies of central memory-derived CD19 CAR T-cell therapy following autologous HSCT in patients with b-cell NHL. Blood (2016) 127(24):2980–90. doi: 10.1182/blood-2015-12-686725 PMC4911862 27118452

[50] Blaeschke F Stenger D Kaeuferle T Willier S Lotfi R Kaiser AD. Induction of a central memory and stem cell memory phenotype in functionally active CD4+ and CD8+ CAR T cells produced in an automated good manufacturing practice system for the treatment of CD19+ acute lymphoblastic leukemia. Cancer Immunol Immunother (2018) 67(7):1053–66. doi: 10.1007/s00262-018-2155-7 PMC11028239 29605883

[51] Bailey SR Nelson MH Majchrzak K Bowers JS Wyatt MM Smith AS. Human CD26high T cells elicit tumor immunity against multiple malignancies via enhanced migration and persistence. Nat Commun (2017) 8(1):1–13. doi: 10.1038/s41467-017-01867-9 29213079 PMC5719008

[52] Rabin M Li M Garforth S Marino J Zheng JH O'Connor KE. Application of novel T cell immunotherapeutics to drive antigen-specific activation, expansion, and differentiation of CD19 chimeric antigen receptor T cells (CAR T-cells). Blood (2020) 136(Supplement 1):34–5. doi: 10.1182/blood-2020-135997

[53] Riddell SR Sommermeyer D Berger C Liu L Balakrishnan A Salter A. Adoptive therapy with chimeric antigen receptor-modified T cells of defined subset composition. Cancer J (2014) 20(2):141–4. doi: 10.1097/PPO.0000000000000036 PMC4149222 24667960

[54] Shifrut E Carnevale J Tobin V Roth TL Woo JM Bui CT. Genome-wide CRISPR screens in primary human T cells reveal key regulators of immune function. Cell (2018) 175(7):1958–1971.e15. doi: 10.1016/j.cell.2018.10.024 30449619 PMC6689405

[55] Eyquem J Mansilla-Soto J Giavridis T Van Der Stegen SJC Hamieh M Cunanan KM. Targeting a CAR to the TRAC locus with CRISPR/Cas9 enhances tumour rejection. Nature (2017) 543(7643):113–7. doi: 10.1038/nature21405 PMC5558614 28225754

[56] Wang Y Tong C Dai H Wu Z Han X Guo Y. Low-dose decitabine priming endows CAR T cells with enhanced and persistent antitumour potential via epigenetic reprogramming. Nat Commun (2021) 12(1):409. doi: 10.1038/s41467-020-20696-x 33462245 PMC7814040

[57] Zhou J Jin L Wang F Zhang Y Liu B Zhao T. Chimeric antigen receptor T (CAR-T) cells expanded with IL-7/IL-15 mediate superior antitumor effects. Protein Cell (2019) 10(10):764–9. doi: 10.1007/s13238-019-0643-y PMC6776495 31250350

[58] Watanabe N Mo F McKenna MK. Impact of manufacturing procedures on CAR T cell functionality. Front Immunol (2022) 13:876339. doi: 10.3389/fimmu.2022.876339 35493513 PMC9043864

[59] Neelapu SS Tummala S Kebriaei P Wierda W Gutierrez C Locke FL. Chimeric antigen receptor T-cell therapy-assessment and management of toxicities. Nat Rev Clin Oncol (2018) 15(1):47–62. doi: 10.1038/nrclinonc.2017.148 28925994 PMC6733403

[60] Rubin DB Danish HH Ali AB Li K LaRose S Monk AD. Neurological toxicities associated with chimeric antigen receptor T-cell therapy. Brain (2019) 142(5):1334–48. doi: 10.1093/brain/awz053 30891590

[61] O'Rourke DM Nasrallah MP Desai A Melenhorst JJ Mansfield K Morrissette JJD. A single dose of peripherally infused EGFRvIII-directed CAR T cells mediates antigen loss and induces adaptive resistance in patients with recurrent glioblastoma. Sci Transl Med (2017) 9(399):eaaa0984. doi: 10.1126/scitranslmed.aaa0984 28724573 PMC5762203

[62] Dholaria BR Dholaria BR Bachmeier CA Bachmeier CA Locke F Locke F. Mechanisms and management of chimeric antigen receptor T-cell therapy-related toxicities. BioDrugs (2019) 33(1):45–60. doi: 10.1007/s40259-018-0324-z 30560413 PMC6733400

[63] Parker KR Migliorini D Perkey E Yost KE Bhaduri A Bagga P. Single-cell analyses identify brain mural cells expressing CD19 as potential off-tumor targets for CAR-T immunotherapies. Cell (Cambridge) (2020) 183(1):126–142.e17. doi: 10.1016/j.cell.2020.08.022 32961131 PMC7640763

[64] Jie J Hao S Jiang S Li Z Yang M Zhang W. Phase 1 trial of the safety and efficacy of fully human anti-bcma CAR T cells in Relapsed/Refractory multiple myeloma. Blood (2019) 134(Supplement_1):4435–5. doi: 10.1182/blood-2019-126104

[65] Gust J Hay KA Hanafi L-A Li D Myerson D Gonzalez-Cuyar LF. Endothelial activation and blood–brain barrier disruption in neurotoxicity after adoptive immunotherapy with CD19 CAR-T cells. Cancer Discovery (2017) 7(12):1404–19. doi: 10.1158/2159-8290.CD-17-0698 PMC5718945 29025771

[66] Safarzadeh Kozani P Safarzadeh Kozani P Rahbarizadeh F Khoshtinat Nikkhoi S. Strategies for dodging the obstacles in CAR T cell therapy. Front Oncol (2021) 11:627549. doi: 10.3389/fonc.2021.627549 33869011 PMC8047470

[67] Mueller KT Waldron E Grupp SA Levine JE Laetsch TW Pulsipher MA. Clinical pharmacology of tisagenlecleucel in b-cell acute lymphoblastic leukemia. Clin Cancer Res (2018) 24(24):6175–84. doi: 10.1158/1078-0432.CCR-18-0758 PMC7433345 30190371

[68] Sterner RM Sakemura R Cox MJ Yang N Khadka RH Forsman CL. GM-CSF inhibition reduces cytokine release syndrome and neuroinflammation but enhances CAR-T cell function in xenografts. Blood (2019) 133(7):697–709. doi: 10.1182/blood-2018-10-881722 30463995 PMC6376281

[69] Zhou X Brenner MK. Improving the safety of T-cell therapies using an inducible caspase-9 gene. Exp Hematol (2016) 44(11):1013–9. doi: 10.1016/j.exphem.2016.07.011 PMC5083205 27473568

[70] Arnold DE Maude SL Callahan CA DiNofia AM Grupp SA Heimall JR. Subcutaneous immunoglobulin replacement following CD19-specific chimeric antigen receptor T-cell therapy for b-cell acute lymphoblastic leukemia in pediatric patients. Pediatr Blood Cancer (2020) 67(3):e28092–n/a. doi: 10.1002/pbc.28092 31793170

[71] Pan J Niu Q Deng B Liu S Wu T Gao Z. CD22 CAR T-cell therapy in refractory or relapsed b acute lymphoblastic leukemia. Leukemia (2019) 33(12):2854–66. doi: 10.1038/s41375-019-0488-7 31110217

[72] Morgan RA Yang JC Kitano M Dudley ME Laurencot CM Rosenberg SA. Case report of a serious adverse event following the administration of T cells transduced with a chimeric antigen receptor recognizing ERBB2. Mol Ther (2010) 18(4):843–51. doi: 10.1038/mt.2010.24 PMC2862534 20179677

[73] Sahin A Sanchez C Bullain S Waterman P Weissleder R Carter BS. Development of third generation anti-EGFRvIII chimeric T cells and EGFRvIII-expressing artificial antigen presenting cells for adoptive cell therapy for glioma. PloS One (2018) 13(7):e0199414–e0199414. doi: 10.1371/journal.pone.0199414 29975720 PMC6033533

[74] Bagley S Desai A Binder Z Nasrallah M Hwang W-T Maloney-Wilensky E. RBTT-12. A phase I study of egfrviii-directed car T cells combined with pd-1 inhibition in patients with newly, diagnosed, mgmt-unmethylated glioblastoma: Trial in progress. Neuro-oncology (2019) 21(Supplement_6):vi221–1. doi: 10.1093/neuonc/noz175.923

[75] He Y Schreiber K Wolf SP Wen F Steentoft C Zerweck J. Multiple cancer-specific antigens are targeted by a chimeric antigen receptor on a single cancer cell. JCI Insight (2019) 4(23):e130416. doi: 10.1172/jci.insight.135306 PMC6962012 31801912

[76] Posey Avery D Schwab Robert D Boesteanu Alina C Steentoft C Mandel U Engels B. Engineered CAR T cells targeting the cancer-associated tn-glycoform of the membrane mucin MUC1 control adenocarcinoma. Immunity (2016) 44(6):1444–54. doi: 10.1016/j.immuni.2016.05.014 PMC5358667 27332733

[77] Zhang Q Liu G Liu J Yang M Fu J Liu G. The antitumor capacity of mesothelin-CAR-T cells in targeting solid tumors in mice. Mol Ther Oncolytics (2021) 20:556–68. doi: 10.1016/j.omto.2021.02.013 PMC7943972 33738341

[78] Haas AR Tanyi JL O’Hara MH Gladney WL Lacey SF Torigian DA. Phase I study of lentiviral-transduced chimeric antigen receptor-modified T cells recognizing mesothelin in advanced solid cancers. Mol Ther (2019) 27(11):1919–29. doi: 10.1016/j.ymthe.2019.07.015 PMC6838875 31420241

[79] Richman SA Nunez-Cruz S Moghimi B Li LZ Gershenson ZT Mourelatos Z. High-affinity GD2-specific CAR T cells induce fatal encephalitis in a preclinical neuroblastoma model. Cancer Immunol Res (2018) 6(1):36–46. doi: 10.1158/2326-6066.CIR-17-0211 29180536 PMC6004321

[80] Hinrichs CS Restifo NP. Reassessing target antigens for adoptive T-cell therapy. Nat Biotechnol (2013) 31(11):999–1008. doi: 10.1038/nbt.2725 24142051 PMC4280065

[81] Safarzadeh Kozani P Safarzadeh Kozani P Rahbarizadeh F. Novel antigens of CAR T cell therapy: New roads old destination. Transl Oncol (2021) 14(7):101079–9. doi: 10.1016/j.tranon.2021.101079 PMC8065293 33862524

[82] Wilkie S van Schalkwyk MCI Hobbs S Davies DM van der Stegen SJC Pereira ACP. Dual targeting of ErbB2 and MUC1 in breast cancer using chimeric antigen receptors engineered to provide complementary signaling. J Clin Immunol (2012) 32(5):1059–70. doi: 10.1007/s10875-012-9689-9 22526592

[83] Roybal Kole T Rupp Levi J Morsut L Walker Whitney J McNally Krista A Park Jason S. Precision tumor recognition by T cells with combinatorial antigen-sensing circuits. Cell (2016) 164(4):770–9. doi: 10.1016/j.cell.2016.01.011 PMC4752902 26830879

[84] Salzer B Schueller CM Zajc CU Peters T Schoeber MA Kovacic B. Engineering AvidCARs for combinatorial antigen recognition and reversible control of CAR function. Nat Commun (2020) 11(1):4166–6. doi: 10.1038/s41467-020-17970-3 PMC7441178 32820173

[85] Seitz CM Kieble V Illi C Reiter S Grote S Mittelstaet J. Combinatorial targeting of multiple shared antigens by adapter-CAR-T cells (aCAR-ts) allows target cell discrimination and specific lysis based on differential expression profiles. Blood (2018) 132(Supplement 1):4543–3. doi: 10.1182/blood-2018-99-115630

[86] Efremova M Finotello F Rieder D Trajanoski Z. Neoantigens generated by individual mutations and their role in cancer immunity and immunotherapy. Front Immunol (2017) 8:1679–9. doi: 10.3389/fimmu.2017.01679 PMC5712389 29234329

[87] Akatsuka Y. TCR-like CAR-T cells targeting MHC-bound minor histocompatibility antigens. Front Immunol (2020) 11:257–7. doi: 10.3389/fimmu.2020.00257 PMC7058980 32184779

[88] Karasaki T Nagayama K Kuwano H Nitadori J Sato M Anraku M. Prediction and prioritization of neoantigens: integration of RNA sequencing data with whole-exome sequencing. Cancer Sci (2017) 108(2):170–7. doi: 10.1111/cas.13131 PMC5329159 27960040

[89] Ali M Foldvari Z Giannakopoulou E Boschen M-L Stronen E Yang W. Induction of neoantigen-reactive T cells from healthy donors. Nat Protoc (2019) 14(6):1926–43. doi: 10.1038/s41596-019-0170-6 31101906

[90] Poorebrahim M Mohammadkhani N Mahmoudi R Gholizadeh M Fakhr E Cid-Arregui A. TCR-like CARs and TCR-CARs targeting neoepitopes: an emerging potential. Cancer Gene Ther (2021) 28:581–9. doi: 10.1038/s41417-021-00307-7 PMC8203496 33654227

[91] Walseng E Köksal H Sektioglu IM Fåne A Skorstad G Kvalheim G. A TCR-based chimeric antigen receptor. Sci Rep (2017) 7(1):10713–10. doi: 10.1038/s41598-017-11126-y PMC5587706 28878363

[92] Oh J Warshaviak DT Mkrtichyan M Munguia ML Lin A Chai F. Single variable domains from the T cell receptor beta chain function as mono- and bifunctional CARs and TCRs. Sci Rep (2019) 9(1):17291–12. doi: 10.1038/s41598-019-53756-4 PMC6872726 31754147

[93] Bijen HM van der Steen DM Hagedoorn RS Wouters AK Wooldridge L Falkenburg JHF. Preclinical strategies to identify off-target toxicity of high-affinity TCRs. Mol Ther (2018) 26(5):1206–14. doi: 10.1016/j.ymthe.2018.02.017 PMC5993934 29567312

[94] Orlando EJ Han X Tribouley C Wood PA Leary RJ Riester M. Genetic mechanisms of target antigen loss in CAR19 therapy of acute lymphoblastic leukemia. Nat Med (2018) 24(10):1504–6. doi: 10.1038/s41591-018-0146-z 30275569

[95] Ruella M Barrett DM Kenderian SS Shestova O Hofmann TJ Perazzelli J. Dual CD19 and CD123 targeting prevents antigen-loss relapses after CD19-directed immunotherapies. J Clin Invest (2016) 126(10):3814–26. doi: 10.1172/JCI87366 PMC5096828 27571406

[96] Ruella M Maus MV. Catch me if you can: Leukemia escape after CD19-directed T cell immunotherapies. Comput Struct Biotechnol J (2016) 14(C):357–62. doi: 10.1016/j.csbj.2016.09.003 PMC5061074 27761200

[97] Hegde M Mukherjee M Grada Z Pignata A Landi D Navai SA. Tandem CAR T cells targeting HER2 and IL13Rα2 mitigate tumor antigen escape. J Clin Invest (2016) 126(8):3036–52. doi: 10.1172/JCI83416 PMC4966331 27427982

[98] Osborne W Marzolini M Tholouli E Ramakrishnan A Bachier CR McSweeney PA. Phase I Alexander study of AUTO3, the first CD19/22 dual targeting CAR T cell therapy, with pembrolizumab in patients with relapsed/refractory (r/r) DLBCL. J Clin Oncol (2020) 38(15_suppl):8001–1. doi: 10.1200/JCO.2020.38.15_suppl.8001

[99] Fry TJ Shah NN Orentas RJ Stetler-Stevenson M Yuan CM Ramakrishna S. CD22-targeted CAR T cells induce remission in b-ALL that is naive or resistant to CD19-targeted CAR immunotherapy. Nat Med (2018) 24(1):20–8. doi: 10.1038/nm.4441 PMC5774642 29155426

[100] Choi BD Yu X Castano AP Bouffard AA Schmidts A Larson RC. CAR-T cells secreting BiTEs circumvent antigen escape without detectable toxicity. Nat Biotechnol (2019) 37(9):1049–58. doi: 10.1038/s41587-019-0192-1 31332324

[101] Cho JH Collins JJ Wong WW. Universal chimeric antigen receptors for multiplexed and logical control of T cell responses. Cell (2018) 173(6):1426–1438.e11. doi: 10.1016/j.cell.2018.03.038 29706540 PMC5984158

[102] Tchou J Zhao Y Levine BL Zhang PJ Davis MM Melenhorst JJ. Safety and efficacy of intratumoral injections of chimeric antigen receptor (CAR) T cells in metastatic breast cancer. Cancer Immunol Res (2017) 5(12):1152–61. doi: 10.1158/2326-6066.CIR-17-0189 PMC5712264 29109077

[103] van Schalkwyk MC Papa SE Jeannon JP Guerrero Urbano T Spicer JF Maher J. Design of a phase I clinical trial to evaluate intratumoral delivery of ErbB-targeted chimeric antigen receptor T-cells in locally advanced or recurrent head and neck cancer. Hum Gene Ther Clin Dev. (2013) 24(3):134–42. doi: 10.1089/humc.2013.144 24099518

[104] Adusumilli PS Cherkassky L Villena-Vargas J Colovos C Servais E Plotkin J. Regional delivery of mesothelin-targeted CAR T cell therapy generates potent and long-lasting CD4-dependent tumor immunity. Sci Trans Med (2014) 6(261):261ra151–261ra151. doi: 10.1126/scitranslmed.3010162 PMC4373413 25378643

[105] Xie JH Nomura N Lu M Chen SL Koch GE Weng Y. Antibody-mediated blockade of the CXCR3 chemokine receptor results in diminished recruitment of T helper 1 cells into sites of inflammation. J Leukoc Biol (2003) 73(6):771–80. doi: 10.1189/jlb.1102573 12773510

[106] Harlin H Yuru M Peterson AC Yuanyuan ZHA Tretiakova M Slingluff C. Chemokine expression in melanoma metastases associated with CD8+ T-cell recruitment. Cancer Res (2009) 69(7):3077–85. doi: 10.1158/0008-5472.CAN-08-2281 PMC3886718 19293190

[107] Wang Y Wang J Yang X Yang J Lu P Zhao L. Chemokine receptor CCR2b enhanced anti-tumor function of chimeric antigen receptor T cells targeting mesothelin in a non-small-cell lung carcinoma model. Front Immunol (2021) 12:628906–6. doi: 10.3389/fimmu.2021.628906 PMC7992009 33777013

[108] Lin Y Yin H An H Zhou C Zhou L Chen S. Chemokine receptor CCR2b expressing anti-Tn-MUC1 CAR-T cells enhanced anti-breast cancer activity. Ann Oncol (2019) 30:xi12. doi: 10.1093/annonc/mdz448.002

[109] Li H Harrison EB Li H Hirabayashi K Chen J Li Q-X. Targeting brain lesions of non-small cell lung cancer by enhancing CCL2-mediated CAR-T cell migration. Nat Commun (2022) 13(1):2154. doi: 10.1038/s41467-022-29647-0 35443752 PMC9021299

[110] Luke JJ Bao R Spranger S Sweis RF Gajewski T. Correlation of WNT/β-catenin pathway activation with immune exclusion across most human cancers. J Clin Oncol (2016) 34(15_suppl):3004–4. doi: 10.1200/JCO.2016.34.15_suppl.3004 PMC6522301 30635339

[111] Fu X Rivera A Tao L Zhang X. An HSV-2 based oncolytic virus can function as an attractant to guide migration of adoptively transferred T cells to tumor sites. Oncotarget (2015) 6(2):902–14. doi: 10.18632/oncotarget.2817 PMC4359264 25460506

[112] Nishio N Diaconu I Liu H Cerullo V Caruana I Hoyos V. Armed oncolytic virus enhances immune functions of chimeric antigen receptor-modified T cells in solid tumors. Cancer Res (2014) 74(18):5195–205. doi: 10.1158/0008-5472.CAN-14-0697 PMC4167556 25060519

[113] Corrales L Matson V Flood B Spranger S Gajewski TF. Innate immune signaling and regulation in cancer immunotherapy. Cell Res (2017) 27(1):96–108. doi: 10.1038/cr.2016.149 27981969 PMC5223230

[114] Ding Q Lu P Xia Y Ding S Fan Y Li X. CXCL9: evidence and contradictions for its role in tumor progression. Cancer Med (2016) 5(11):3246–59. doi: 10.1002/cam4.934 PMC5119981 27726306

[115] Ding Q Xia Y Ding S Lu P Sun L Liu M. An alternatively spliced variant of CXCR3 mediates the metastasis of CD133+ liver cancer cells induced by CXCL9. Oncotarget (2016) 7(12):14405–14. doi: 10.18632/oncotarget.7360 PMC4924724 26883105

[116] Korniejewska A McKnight AJ Johnson Z Watson ML Ward SG. Expression and agonist responsiveness of CXCR3 variants in human T lymphocytes. Immunology (2011) 132(4):503–15. doi: 10.1111/j.1365-2567.2010.03384.x PMC3075504 21255008

[117] Grosskopf AK Labanieh L Klysz DD Roth GA Xu P Adebowale O. Delivery of CAR-T cells in a transient injectable stimulatory hydrogel niche improves treatment of solid tumors. Sci Adv (2022) 8(14):eabn8264. doi: 10.1126/sciadv.abn8264 35394838 PMC8993118

[118] Zeng B Middelberg APJ Gemiarto A MacDonald K Baxter AG Talekar M. Self-adjuvanting nanoemulsion targeting dendritic cell receptor Clec9A enables antigen-specific immunotherapy. J Clin Invest (2018) 128(5):1971–84. doi: 10.1172/JCI96791 PMC5919883 29485973

[119] Risom L Møller P Loft S. Oxidative stress-induced DNA damage by particulate air pollution. Mutat Res (2005) 592(1-2):119–37. doi: 10.1016/j.mrfmmm.2005.06.012 16085126

[120] Birbrair A. Tumor microenvironment extracellular matrix components. part a. Cham: Springer (2020).

[121] Caruana I Savoldo B Hoyos V Weber G Liu H Kim ES. Heparanase promotes tumor infiltration and antitumor activity of CAR-redirected T lymphocytes. Nat Med (2015) 21(5):524–9. doi: 10.1038/nm.3833 PMC4425589 25849134

[122] Chen Q Hu Q Dukhovlinova E Chen G Ahn S Wang C. Photothermal therapy: Photothermal therapy promotes tumor infiltration and antitumor activity of CAR T cells (Adv. mater. 23/2019). Advanced materials (Weinheim) (2019) 31(23):1970166–n/a. doi: 10.1002/adma.201900192 PMC7262962 30916367

[123] Lim AR Rathmell WK Rathmell JC. The tumor microenvironment as a metabolic barrier to effector T cells and immunotherapy. Elife (2020) 9. doi: 10.7554/eLife.55185 PMC7200151 32367803

[124] Kosti P Opzoomer JW Larios-Martinez KI Henley-Smith R Scudamore CL Okesola M. Hypoxia-sensing CAR T cells provide safety and efficacy in treating solid tumors. Cell Rep Med (2021) 2(4):100227. doi: 10.1016/j.xcrm.2021.100227 33948568 PMC8080111

[125] Oh SA Li MO. TGF-β: Guardian of T cell function. J Immunol (2013) 191(8):3973–9. doi: 10.4049/jimmunol.1301843 PMC3856438 24098055

[126] Kloss CC Lee J Zhang A Chen F Melenhorst JJ Lacey SF. Dominant-negative TGF-β receptor enhances PSMA-targeted human CAR T cell proliferation and augments prostate cancer eradication. Mol Ther (2018) 26(7):1855–66. doi: 10.1016/j.ymthe.2018.05.003 PMC6037129 29807781

[127] Tang N Cheng C Zhang X Qiao M Li N Mu W. TGF-beta inhibition via CRISPR promotes the long-term efficacy of CAR T cells against solid tumors. JCI Insight (2020) 5(4). doi: 10.1172/jci.insight.133977 PMC7101140 31999649

[128] Avanzi MP Yeku O Li X Wijewarnasuriya DP van Leeuwen DG Cheung K. Engineered tumor-targeted T cells mediate enhanced anti-tumor efficacy both directly and through activation of the endogenous immune system. Cell Rep (2018) 23(7):2130–41. doi: 10.1016/j.celrep.2018.04.051 PMC5986286 29768210

[129] Adachi K Kano Y Nagai T Okuyama N Sakoda Y Tamada K. IL-7 and CCL19 expression in CAR-T cells improves immune cell infiltration and CAR-T cell survival in the tumor. Nat Biotechnol (2018) 36(4):346–51. doi: 10.1038/nbt.4086 29505028

[130] Yeku OO Purdon TJ Koneru M Spriggs D Brentjens RJ. Armored CAR T cells enhance antitumor efficacy and overcome the tumor microenvironment. Sci Rep (2017) 7(1):10541–1. doi: 10.1038/s41598-017-10940-8 PMC5585170 28874817

[131] Zhang F Stephan SB Ene CI Smith TT Holland EC Stephan MT. Nanoparticles that reshape the tumor milieu create a therapeutic window for effective t-cell therapy in solid malignancies. Cancer Res (2018) 78(13):3718–30. doi: 10.1158/0008-5472.CAN-18-0306 PMC6030470 29760047

[132] Gargett T Yu W Dotti G Yvon ES Christo SN Hayball JD. GD2-specific CAR T cells undergo potent activation and deletion following antigen encounter but can be protected from activation-induced cell death by PD-1 blockade. Mol Ther (2016) 24(6):1135–49. doi: 10.1038/mt.2016.63 PMC4923328 27019998

[133] Cherkassky L Morello A Villena-Vargas J Feng Y Dimitrov DS Jones DR. Human CAR T cells with cell-intrinsic PD-1 checkpoint blockade resist tumor-mediated inhibition. J Clin Invest (2016) 126(8):3130–44. doi: 10.1172/JCI83092 PMC4966328 27454297

[134] Hu Q Li H Archibong E Chen Q Ruan H Ahn S. Inhibition of post-surgery tumour recurrence via a hydrogel releasing CAR-T cells and anti-PDL1-conjugated platelets. Nat BioMed Eng (2021) 5:1038–47. doi: 10.1038/s41551-021-00712-1 PMC9102991 33903744

[135] Labanieh L Majzner RG Klysz D Sotillo E Fisher CJ Vilches-Moure JG. Enhanced safety and efficacy of protease-regulated CAR-T cell receptors. Cell (2022) 185(10):1745–1763.e22. doi: 10.1016/j.cell.2022.03.041 35483375 PMC9467936

[136] Giuffrida L Sek K Henderson MA Lai J Chen AXY Meyran D. CRISPR/Cas9 mediated deletion of the adenosine A2A receptor enhances CAR T cell efficacy. Nat Commun (2021) 12(1):3236. doi: 10.1038/s41467-021-23331-5 34050151 PMC8163771

[137] Larson RC Kann MC Bailey SR Haradhvala NJ Llopis PM Bouffard AA. CAR T cell killing requires the IFNγR pathway in solid but not liquid tumours. Nature (2022) 604(7906):563–70. doi: 10.1038/s41586-022-04585-5 35418687

[138] Legut M Gajic Z Guarino M Daniloski Z Rahman JA Xue X. A genome-scale screen for synthetic drivers of T cell proliferation. Nature (2022) 603(7902):728–35. doi: 10.1038/s41586-022-04494-7 PMC9908437 35296855

[139] Ye L Park JJ Peng L Yang Q Chow RD Dong MB. A genome-scale gain-of-function CRISPR screen in CD8 T cells identifies proline metabolism as a means to enhance CAR-T therapy. Cell Metab (2022) 34(4):595–614.e14. doi: 10.1016/j.cmet.2022.02.009 35276062 PMC8986623

[140] Gattinoni L Speiser DE Lichterfeld M Bonini C. T Memory stem cells in health and disease. Nat Med (2017) 23(1):18–27. doi: 10.1038/nm.4241 28060797 PMC6354775

[141] Ghorani E Reading JL Henry JY Massy MRd Rosenthal R Turati V. The T cell differentiation landscape is shaped by tumour mutations in lung cancer. Nat Cancer (2020) 1(5):546–61. doi: 10.1038/s43018-020-0066-y PMC7115931 32803172

[142] Biasco L Izotova N Rivat C Ghorashian S Richardson R Guvenel A. Clonal expansion of T memory stem cells determines early anti-leukemic responses and long-term CAR T cell persistence in patients. Nat Cancer (2021) 2(6):629–42. doi: 10.1038/s43018-021-00207-7 PMC7611448 34345830

[143] Murray C Pao E Jann A Park DE Di Carlo D. Continuous and quantitative purification of T-cell subsets for cell therapy manufacturing using magnetic ratcheting cytometry. SLAS Technol (2018) 23(4):326–37. doi: 10.1177/2472630317748655 PMC6092733 29281498

[144] Shukla S Langley MA Singh J Edgar JM Mohtashami M Zúñiga-Pflücker JC. Progenitor T-cell differentiation from hematopoietic stem cells using delta-like-4 and VCAM-1. Nat Methods (2017) 14(5):531–8. doi: 10.1038/nmeth.4258 28394335

[145] Trotman-Grant AC Mohtashami M De Sousa Casal J Martinez EC Lee D Teichman S. DL4-μbeads induce T cell lineage differentiation from stem cells in a stromal cell-free system. Nat Commun (2021) 12(1):5023–3. doi: 10.1038/s41467-021-25245-8 PMC8373879 34408144

